# The Salem simulator version 2.0: a tool for predicting the productivity of pure and mixed stands and simulating management operations

**DOI:** 10.12688/openreseurope.13671.1

**Published:** 2021-06-04

**Authors:** Raphaël Aussenac, Thomas Pérot, Mathieu Fortin, Francois de Coligny, Jean-Matthieu Monnet, Patrick Vallet

**Affiliations:** 1 Univ. Grenoble Alpes, INRAE, LESSEM, 2 rue de la Papeterie-BP 76, St-Martin-d'Hères, F-38402, France; 2 INRAE, EFNO, Domaine des Barres, Nogent-sur-Vernisson, F-45290, France; 3 Natural Resources Canada, Canadian Forest Service, Canadian Wood Fibre Centre, 580 Booth Str., Ottawa, Ontario, Canada; 4 AMAP, Univ Montpellier, CIRAD, CNRS, INRAE, IRD, Montpellier, 34000, France

**Keywords:** forest, growth model, mixture effect, bark model, allometry, diameter distribution, circumference-height relationship, volume equation

## Abstract

A growing body of research suggests mixed-species stands are generally more productive than pure stands. However, this effect of mixture depends on species assemblages and environmental conditions and forest managers often lack tools to assess the potential benefit of shifting from pure to mixed stands. Here we present Salem, a simulator filling this gap. Salem predicts the dynamics of pure and mixed even-aged stands and makes it possible to simulate management operations. Its purpose is to be a decision support tool for forest managers and stakeholders as well as for policy makers. It is also designed to conduct virtual experiments and help answer research questions.

In Salem, we parameterised the growth in pure stand of 12 common tree species of Europe and we assessed the effect of mixture on species growth for 24 species pairs (made up of the 12 species mentioned above). Thus, Salem makes it possible to compare the productivity of 36 different pure and mixed stands depending on environmental conditions and user-defined management strategies. Salem is essentially based on the analysis of National Forest Inventory data. A major outcome of this analysis is that we found species mixture most often increases species growth, in particular at the poorest sites. Independently from the simulator, foresters and researchers can also consider using the species-specific models that constitute Salem: the growth models including or excluding mixture effect, the bark models, the diameter distribution models, the circumference-height relationship models, as well as the volume equations for the 12 parameterised species. Salem runs on Windows, Linux, or Mac. Its user-friendly graphical user interface makes it easy to use for non-modellers. Finally, it is distributed under a LGPL license and is therefore free and open source.

## 1 Introduction

Over the last decades, mixed-species stands have received growing attention, in particular because of their higher productivity compared to pure stands ( Ammer, 2019; Liang *et al*., 2016). However, this effect of mixture is highly variable and depends on species and environmental conditions ( Paquette & Messier, 2011; Toïgo *et al*., 2015). As a result, forest managers often lack information to consider mixed stands as a possible alternative to pure stands. Forest dynamics models ( Pretzsch, 2009; Weiskittel *et al*., 2011) can help fill this information gap.

At present, numerous models simulate mixed stand management and dynamics (see for example Courbaud *et al*., 2015; Lasch-Born *et al*., 2020; Schumacher *et al*., 2006; Wernsdörfer *et al*., 2012). Four different approaches are used to represent mixed stand productivity ( Pretzsch *et al*., 2015): (1) by deriving their productivity as a weighted mean of the productivity of their constituent species using species-specific models; (2) by integrating species-specific growing space competition indices in individual-tree growth models; (3) by using empirical multipliers affecting growth rates and stand density depending on species assemblages and environmental conditions; and (4) via a process-based approach by incorporating within-stand environmental conditions, species-specific structures, and resource uptake and availability. Models simulating mixed stand management and dynamics also differ in the type of calibration data they require and in the spatial scale at which they operate (tree, stand, landscape). These different calibration data, operation scales and ways of representing mixed stand productivity each have their advantages, but they are not all of equal interest to forest managers.

With a view to being used by forest managers, models using the multiplier approach (3) have an advantage: their generally simpler functioning makes them less data-and prescription-intensive. On the other hand, models based on approach (2) and (4) require fine-scale spatial data that forest managers are unlikely to have. As for approach (1), it does not take into account species interactions and can therefore only partially render the mixture effect on stand productivity.

Data from national forest inventories (NFI) are particularly relevant for modelling pure and mixed stand dynamics with a view to assisting forest managers. The wide geographical coverage of these data makes it possible to study the dynamics of a large variety of pure and mixed stands under diverse environmental and silvicultural conditions (e.g., Fortin & Langevin, 2012; Lessard *et al*., 2001; Li *et al*., 2011). Such datasets make it possible, in particular, to compare the dynamics of pure and mixed stands and thus to estimate the effect of mixture on stand productivity for different species assemblages, while controlling for the effect of environmental conditions on this composition - productivity relationship. NFI data are therefore suitable for modelling various stands in a variety of contexts, which is useful for managers looking to compare different strategies before defining a management strategy. Conversely, experimental plots and smaller plot networks are generally less capable of producing reliable simulations in different contexts due to their limited coverage of environmental gradients, species assemblages and management practices. Their usefulness lies more in their contribution to the understanding of the mechanisms underlying stand dynamics.

Models with a stand-level approach ( Pretzsch, 2009; Weiskittel *et al*., 2011) are particularly convenient for forest managers, and especially for even-aged stand managers, as they are in line with the spatial scale at which management is usually planned. Stand-level models run with data that forest managers are likely to have, such as mean diameter or total basal area, and management strategies can be prescribed as in a traditional management plan by formulating stand-level prescriptions defining, for example, a thinning intensity or a target mean diameter. On the other hand, landscape models are too large in scale to plan silvicultural operations, while tree-level models can be too data- or prescription-intensive. Landscape models are more relevant for large-scale spatial planning, while tree-level models can be more relevant for studying interactions among trees, for example.

Here we present the Salem (for StAnd LEvel Model) simulator that was developed to best meet the needs of forest managers. Salem predicts the dynamics of even-aged pure and mixed stands and makes it possible to simulate management operations. Its purpose is to be a decision support tool for forest managers and stakeholders as well as for policy makers. Salem is calibrated on stand-level data from the French National Forest Inventory (NFI) as well as on environmental data and it simulates stand-level processes. The mixture effect on stand productivity is explicitly implemented in Salem using the multiplier approach (3) mentioned above. To date, this approach has been adopted very little in stand-level models despite its advantage over the other approaches (but see: Vospernik, 2021). Altogether, 12 common tree species of Europe are parameterised, making it possible to simulate the same number of pure stands and 24 different bispecific mixed stands corresponding to frequently observed assemblages of these 12 species. In France, these 12 species represent 72% of the total volume of wood available in the production forests, excluding poplar stands ( IGN, 2020). Salem is distributed under a LGPL license and is therefore free and open source.

Salem has been developed for more than 10 years through several conventions and projects (see Grant information). In earlier versions Salem has already proven to be a valuable tool to plan forest management but also to better understand the functioning of forest ecosystems. Salem is now a mature and reliable tool ready to be used. We present its general functioning in Section 2. We then provide details on the various used models in Section 3. Finally, we show how to use Salem and how to implement management operations in Section 4.

## 2 Implementation

**Figure 1.**
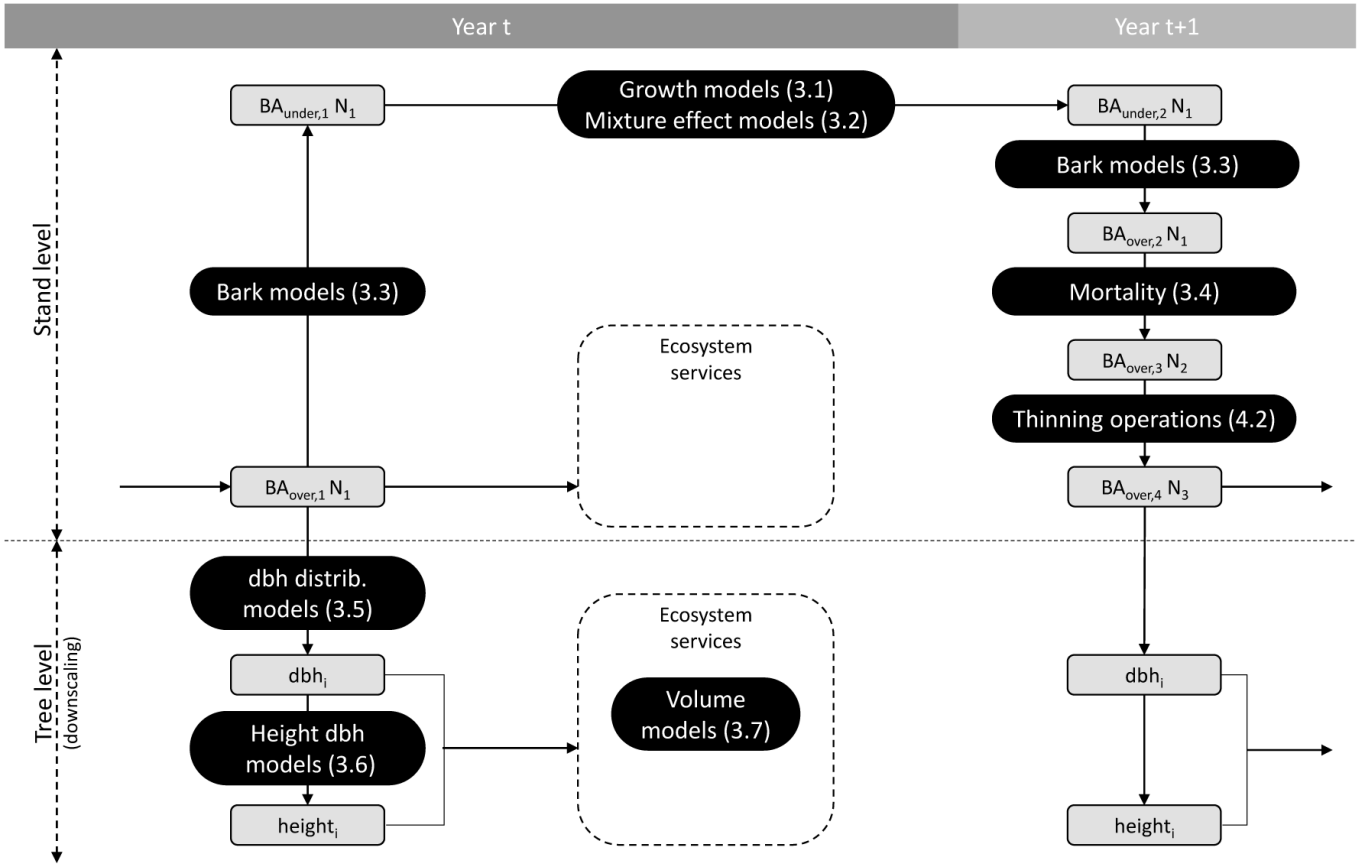
General functioning of Salem. Grey boxes indicate the state of the following variables: over or under bark basal area (BA), number of trees (N), tree diameter at breast height (dbh), and tree height. Black boxes indicate the models used to change variables from one state to another. Numbers in brackets correspond to the section numbers in which the models are presented.

Salem consists of different models chained together to simulate forest dynamics and management operations. The general functioning of Salem is summarised in Figure 1, which shows how the different models are connected to each other.

In the first instance, Salem requires the stand initial state as input, *i.e.* the basal area (BA), the mean quadratic diameter (Dg), and the site indices for one or two species (for mono- or bi-specific stands). In Salem, site indices are calculated from a species-specific combination of environmental variables ( Section 3.1). Salem then calculates the under bark species BA using species-specific bark models ( Section 3.3). Thereafter, Salem calculates the under bark stand basal area increment (BAI) using species-specific growth models ( Section 3.1). If the simulated stand is composed of two species, the effect of each species on the other species growth is simulated ( Section 3.2). Next, the stand under bark BA is converted back into over bark BA. At that stage, if the simulated stand density exceeds the self-thinning boundary, mortality is triggered and a certain amount of BA is removed to bring back the stand below this boundary ( Section 3.4). Finally, thinning operations can be simulated by removing a certain amount of BA per species ( Section 4). Salem operates on an annual time step and the procedure described above is repeated for each simulated year.

At each annual time step stand level variables can be downscaled to tree level variables. Salem uses species-specific models to generate tree diameter distributions from the stand level values Dg and N of each species ( Section 3.5). From these predicted diameters as well as from environmental and stand variables, tree height is predicted ( Section 3.6). Finally, a two-entry (diameter and height) volume equation is applied to predict tree volume ( Section 3.7).

The modelling framework presented here is the result of successive developments, some of which have been presented in previous studies. Table 1 summarises the differences between previous work and the work presented in this article.

**Table 1.** Differences between previous work and the work presented in this article. ^a ^the self-thinning boundary is used to calculate the pure and mixed stands density indices (DI) and to trigger mortality.

	Already published	Published here
Growth models in pure stands	Method: Vallet & Pérot (2011) Model calibrated on five species: Toïgo *et al*. (2015) with data from NFI 2006–2010 Model calibrated on two species: Vallet & Perot (2018) with data from NFI 2006–2012	Models (re)calibrated on 12 species with data from NFI 2006–2013
Models of the mixture effect on growth	Method: Vallet & Pérot (2011) Model calibrated on five species pairs: Toïgo *et al*. (2015) with data from NFI 2006– 2010	Models (re)calibrated on 25 species pairs with data from NFI 2006–2013
Models of bark proportion		Models calibrated on nine species with data from Bouvet & Deleuze (2013)
Self-thinning boundary ^a^ and mortality	Method and calibration: Toïgo *et al*. (2018) (appendices) with data from NFI 2006–2012	Models recalibrated with more stands with data from NFI 2006–2012
Models of individual tree diameter distribution		Models calibrated on 12 species with data from NFI 2006–2013
Models of individual tree height		Models calibrated on 12 species with data from NFI 2006–2013
Models of individual tree volume		Models calibrated on 30 species with data from Deleuze *et al*. (2013)

## 3 Model descriptions

### 3.1 Growth models in pure stands

***3.1.1 Data*.** We calibrated our growth models using French NFI data collected between 2006 and 2013 in pure stands according to the following criteria:

- Stands had to be regular stands, as defined by the French NFI.
- Stands must have not been logged in the five years preceding the measurements.
- A single species was to make up the total stands BA.

French NFI data are collected on temporary plots that are measured only once. Trees equal or greater than 7.5 cm in diameter at breast height (dbh) are identified and measured. Tree radial growth in the five years preceding their measurement is assessed using tree cores. In order to develop a predictive growth model, we calculated the stand dendrometric values BA and Dg at the beginning of the five-year growing periods using the diameter measurements and the radial growth measures.

All pure stands were considered, regardless of their location. However, we removed all pure *P. pinaster* stands with a Dg at the beginning of the five-year growing periods smaller than 5 cm. These stands are the result of two major storms: Martin (1999) and Klaus (2009) and have distinctive dynamics. In fact, *P. pinaster* has strong juvenile dynamics, and due to the reconstruction of initial dendrometric values and the minimum inventory dbh of 7.5 cm, stands with very small Dg were only very productive ones, which unbalanced the data. The problem did not appear for other species. We also removed stands with missing environmental variables. Finally, we only modelled the growth of species that were found on at least 100 plots. Table 2 presents the number of inventory plots in pure stands we obtained for those species.

**Table 2.** Number and features of inventory plots in pure stands for each species. Only species found on at least 100 plots are considered. Plots nb is the number of NFI plots used for the models calibration; Nb stems is the mean number of stems per hectare of the plots; Dg is the mean quadratic diameter in cm; T is the mean annual temperature in °C over the 1981–2010 period and P is the annual sum of precipitation in mm over the 1981–2010 period. The climate data come from the French national meteorological service and were processed following the Aurelhy method ( Benichou & Le Breton, 1986). Column Sp indicates the abbreviations of species names used throughout this article.

		Plots	Nb stems	Dg		T		P	
Species	Sp	nb	mean	min	max	min	max	min	max
*Quercus robur*	*Qu. ro.*	489	332	2.6	106.5	6.9	14.3	612	1836
*Quercus petraea*	*Qu. pe.*	612	429	1.2	87.7	6.4	13.5	584	1741
*Quercus pubescens*	*Qu. pu.*	229	545	2.4	64.5	8.0	13.8	613	1607
*Fagus sylvatica*	*Fa. sy.*	553	387	2.2	117.0	5.8	13.6	641	2532
*Pinus pinaster*	*Pi. pi.*	1150	514	5.1	66.8	10.7	15.2	598	1526
*Pinus sylvestris*	*Pi. sy.*	615	636	0.8	59.0	5.9	13.4	636	2088
*Pinus nigra* subsp. *laricio*	*Pi. la.*	225	723	0.5	78.8	7.8	13.7	603	1687
*Pinus nigra* subsp. *nigra*	*Pi. ni.*	157	753	2.5	48.3	7.3	14.2	632	1469
*Pinus halepensis*	*Pi. ha.*	162	453	2.8	56.2	11.7	15.3	483	970
*Abies alba*	*Ab. al.*	262	483	3.5	87.0	5.4	11.2	771	2029
*Picea abies*	*Pi. ab.*	526	745	1.5	67.0	3.3	12.9	676	2622
*Pseudotsuga menziesii*	*Ps. me.*	542	518	1.3	61.2	6.5	12.9	630	2331

***3.1.2 Models*.** We modelled species growth in pure stand with a multiplicative model where species potential growth, estimated by a set of environmental variables (X _m_), is limited by the stage of development of the stand, estimated by its mean quadratic diameter (Dg) and by a stand density index (DI). The generic form of our growth model is given by:

$$\;BAI_{ik}=f_{1,i}(X_{m}).f_{2,i}(DI_{k})\;.\;f_{3,i}(Dg_{ik})+{}_{ik}\;(1)$$

where BAI _ik_ is the observation k of the basal area increment per hectare of species i over five years (in m ^2^.ha ^−1^.yr ^−1^), X _m_ is a species-specific combination of environmental variables, DI is the stand density index (see Equation 6), Dg is the mean quadratic diameter (in cm) and is the model residuals assumed to be normally distributed and independent. Dendrometric variables (DI and Dg) are those estimated at the beginning of the five-year growth period.

The *f*_1_ function represents species potential growth and therefore corresponds to a species-specific site index (SI). It is given by:

$$\;f_{1,i}(X_{m})=a_{0,i}+\sum_{m=1}^{n}\left(a_{m,i}.X_{m}\right)=SI_{i}\;(2)$$

where SI _i_ is the site index of species i; and a _0_ and a _m_ are parameters to be estimated. We chose to work with these site indices integrating environmental variables rather than with the site indices commonly used by foresters (defined by the dominant height of stands at a given age) as they are better adapted to describe or predict species growth over wide gradients of environmental conditions ( Bontemps & Bouriaud, 2014).

The *f*_2_ and *f*_3_ functions are detailed in Equation 3 and Equation 4. We retained two forms for the density related reduction function ( *f*_2_), leading to the following two equations:

$$BAI_{ik}=\left(a_{0,i}+\sum_{m=1}^{n}\left(a_{m,i}.X_{m,k}\right)\right)\;.DI_{k}^{b_{i}}.\;\left(\frac{e^{\left(c_{1,i}.Dg_{ik}\right)}+c_{2,i}}{1+c_{2,i}}\right)+{}_{ik}\;(3)$$

$$BAI_{ik}=\left(a_{0,i}+\sum_{m=1}^{n}\left(a_{m,i}.X_{m,k}\right)\right)\;.\;\left(\frac{(1+b_{i}).DI_{k}}{b_{i}+DI_{k}}\right)\;.\;\left(\frac{e^{\left(c_{1,i}.Dg_{ik}\right)}+c_{2,i}}{1+c_{2,i}}\right)+{}_{ik}\;(4)$$

where a _0_, a _m_, b, c _1_ and c _2_ are parameters to be estimated. The *f*_2_ and *f*_3_ functions were chosen according to scatter plot analyses described in Toïgo *et al*. (2015) appendices.

Each species was assigned the model that best fitted its data, *i.e.* the model with the lower Akaike information criterion value (AIC, Akaike, 1974). We also selected environmental variables to be included in X _m_ according to the AIC. We used Equation 4 to model *Abies alba* and *Pseudotsuga menziesii* growth and Equation 3 to model the growth of all other species. To account for heteroscedasticity, a variance function based on the power of the fitted values was included in the model ( Pinheiro & Bates, 2006). Models were fitted using the *gnls* function from the *nlme R* package ( Pinheiro *et al*., 2020). The general fit statistics of the models as well as the environmental variables and their associated parameter estimates are presented in Table 4.

The stand density index DI is calculated independently from the growth model. It is defined relative to the species-specific self-thinning boundary which indicates for all values of Dg the maximum number of trees (N _max_) above which some individuals may die. N _max_ is given by:

$$\;N_{max}=e^{p+q.log(Dg)}\;(5)$$

where p and q are species-specific parameters which estimates are presented in Table 3. We fitted p and q using quantile regressions following the method presented in Toïgo *et al*. (2018).

**Table 3.** Parameter estimates of the self-thinning boundary. Values presented in this table differ from those presented in Toïgo *et al*. (2018) because here the self-thinning boundary is fitted over a larger number of NFI plots (Nb of pure plots). Correspondence between species full names and their abbreviations is presented in Table 2.

Species	Intercept (p)	Slope (q)	Nb of pure plots
*Qu. ro.*	12.60	-1.86	424
*Qu. pe.*	13.10	-2.01	494
*Qu. pu.*	11.98	-1.66	186
*Fa. sy.*	13.99	-2.18	517
*Pi. pi.*	12.48	-1.80	946
*Pi. sy.*	13.44	-2.03	544
*Pi. la.*	10.66	-1.14	155
*Pi. ni.*	12.90	-1.81	111
*Pi. ha.*	12.83	-1.92	135
*Ab. al.*	13.08	-1.86	261
*Pi. ab.*	12.88	-1.76	464
*Ps. me.*	11.15	-1.30	425

**Table 4.** Parameter estimates of pure stand growth models for each species. ^*a*^ calculated from aspect (in degrees) as follows: *cos(aspect* * 2 *π*/360); ^*b*^ soil water holding capacity; ^*c*^ ecological regions. Monthly temperature and potential evapotranspiration (PET) values are 30-year means calculated over the 1981–2010 period. The climate data come from the French national meteorological service and were processed following the Aurelhy method ( Benichou & Le Breton, 1986). Soil water holding capacity (SWHC) is calculated using NFI soil description and Piedallu *et al.* (2018) method. pH and C/N ratio are bioindicated from NFI flora description ( Gégout *et al.*, 2005). All other variables come from the NFI service. Correspondence between species full names and their abbreviations is presented in Table 2.

	Unit	Param.	*Qu. ro.*	*Qu. pe.*	*Qu. pu.*	*Fa. sy.*	*Pi. pi.*	*Pi. sy.*	*Pi. la.*	*Pi. ni.*	*Pi. ha.*	*Ab. al.*	*Pi. ab.*	*Ps. me.*
Equation			3	3	3	3	3	3	3	3	3	4	3	4
Power of variance model			0.912	1.047	0.906	0.817	0.942	0.823	0.762	1.015	0.826	0.771	0.951	0.892
Std dev of residual error (m ^2^/ha/5yrs)			0.434	0.342	0.388	0.421	0.379	0.456	0.582	0.424	0.577	0.535	0.363	0.343
Intercept	-	a _0_	22.00	55.87	6.95	39.71	117.64	291.25	73.41	199.47	19.30	-7.43	94.06	71.36
Elevation	m					-0.0083					-0.0108			
Aspect (North) ^*a*^	-						-4.369						-3.705	
Mask	gr									-0.549				-0.277
Feb. min T°	°C												5.566	
May min T°	°C													-4.301
Dec. min T°	°C			4.372										
Feb. mean T°	°C		0.790											
July max T°	°C							-3.302		-3.593				
June PET	mm											0.446		
May water budget	mm												0.179	
May water deficit	mm		-0.171											
June water deficit	mm						-0.374							
July water deficit	mm			-0.354										
May available water	mm								0.275					
SWHC ^*b*^	mm		0.0282	0.0372	0.0305	0.0531								
SWHC ^*b*^ 1st horiz.	mm											0.5948	1.2870	
pH	-	a _m_						-23.311						2.941
pH ^2^	-							1.359						
C/N	-		-0.384	-0.592	-0.167	-0.417	-1.383	-4.455	-1.020	-1.621		-0.891	-0.938	
C/N ^2^	-							0.066						
Limestone bedrock	Boul.		-3.358	-11.097			-32.884						-8.100	
Hydromorphic soils	Boul.								-8.713					
Weakly hydromorphic soils	Boul.				2.426		6.394							
Carbonate rock	Boul.					2.625								
Brown soil	Boul.				0.840									
Rock outcrop	[0–10]						-1.403							
Rock content 40cm	[0–10]							-0.931	-2.705					
Slope	%		-0.079			-0.086	-0.197	-0.110		-0.350			-0.255	
GRECO A ^*c*^	Boul.											17.83		
GRECO B ^*c*^	Boul.				3.54	4.64								
GRECO C ^*c*^	Boul.							15.37						
GRECO D ^*c*^	Boul.			-7.94										
GRECO H ^*c*^	Boul.							-7.52				-11.56		
GRECO I ^*c*^	Boul.													-26.72
GRECO J ^*c*^	Boul.						-9.6825							
Density (RDI)		b	0.588	0.625	0.631	0.585	0.699	0.591	0.603	0.693	0.600	0.234	0.637	0.450
Mean diameter (Dg)		c _1_	-0.078	-0.111	-0.080	-0.083	-0.106	-0.146	-0.165	-0.158	-0.118	-0.077	-0.129	-0.157
Mean diameter (Dg)		c _2_	0.098	0.063	0.345	0.073	0.051	0.048	0.092	0.058	0.169	0.119	0.104	0.180

DI is then given by:

$$\;DI=\frac{N}{N_{max}}\;(6)$$

where N is the number of stems and N _max_ is the maximum number of trees given by the self-thinning boundary for the corresponding Dg. DI is therefore expected to range between 0 and 1. In Salem, when DI reaches 1, *i.e.* when N reaches N _max_, mortality is triggered (see Section 3.4). This DI has been commonly used in pure stands ( Charru *et al*., 2012; Reineke, 1933) but also in mixed stands ( Del Río *et al*., 2016).

### 3.2 Models of the mixture effect on growth

***3.2.1 Data*.** We modelled the effect of mixture on stand growth using French NFI data collected in mixed stands according to the following criteria:

- Stands had to be regular stands, as defined by the French NFI.
- Stands must have not been logged in the five years preceding the measurements.
- The sum of the BA of the two species had to account for at least 80% of the total BA. In addition, each of the two species had to account for a greater proportion of the total BA than all the remaining species combined.
- In our approach, we compared the observed growth of mixed stands to their expected growth given by the models in pure stands ( Vallet & Pérot, 2011). To avoid using our pure stand growth models outside their geographical range of validity, we only considered mixed stands in sylvoécorégions (SER: homogeneous areas defined by the French NFI service; Inventaire-Forestier-National, 2011) where at least five pure stands were found for the adjustment of the growth models of the corresponding species.

Table 5 presents the number of inventory plots in mixed stands we obtained. We analysed the effect of mixture only for species pairs found on at least 20 plots. Altogether, 24 species pairs were studied.

**Table 5.** Number of inventory plots for each species pair. Only species pairs found on at least 20 plots (in bold) were considered. Correspondence between species full names and their abbreviations is presented in Table 2.

	*Qu. ro.*	*Qu. pe.*	*Qu. pu.*	*Fa. sy.*	*Pi. pi.*	*Pi. sy.*	*Pi. la.*	*Pi. ni.*	*Pi. ha.*	*Ab. al.*	*Pi. ab.*
*Qu. pe.*	**185**										
*Qu. pu.*	**61**	15									
*Fa. sy.*	**174**	**561**	6								
*Pi. pi.*	**106**	15	6	0							
*Pi. sy.*	**79**	**86**	**81**	**150**	3						
*Pi. la.*	14	11	2	7	**25**	**23**					
*Pi. ni.*	7	7	11	9	0	**77**	0				
*Pi. ha.*	0	0	17	0	0	0	0	1			
*Ab. al.*	2	**24**	0	**284**	0	**56**	0	1	0		
*Pi. ab.*	**29**	19	0	**126**	0	**79**	2	6	0	**278**	
*Ps. me.*	**23**	**24**	1	15	0	**34**	3	1	0	**68**	**63**

***3.2.2 Modelling of the mixture effect*.** The effect of mixture is defined here as the relative difference between the observed productivity of a species in a mixed stand and its expected productivity if it was in pure stand. It is calculated as follows:

$$\;u_{ij}=\frac{BAI_{ij}-E_{i}}{E_{i}}\;(7)$$

where u _ij_ is the mixture effect on the basal area increment of species i when mixed with species j; BAI _ij_ is the observed basal area increment of species i when mixed with species j; and E _i_ is the expected basal area increment of species i in a mixed stand predicted from its productivity in pure stand and weighted by its proportion in the mixed stand. E _i_ is therefore given by:

$$\;E_{i}=x_{i}.\hat{BAI_{i}}\;(8)$$

where $\hat{BAI_{i}}$ is the basal area increment of species i predicted from pure stand growth models ( Equation 3 and Equation 4); and x _i_ is the mixture proportion, calculated as the ratio of species i density index (DI _i_) over the total density (DI). In mixed stands, the total DI is calculated as the sum of the species partial densities (DI _i_) following the method presented in Del Río *et al*. (2016). In mixed stands DI is thus given by:

$$\;DI=\sum_{i}DI_{i}=\sum_{i}\frac{N_{i}}{e^{p+q.log(Dg_{i})}}\;(9)$$

where N _i_ is the number of trees of species i and DG _i_ is species i mean quadratic diameter; p and q are the same parameters as those used in Equation 5, which estimates are presented in Table 3.

After calculating the mixture effects u _ij_ (with Equation 7) for all plots, we modelled it as a function of mixture proportion as follows:

$$\;u_{ijk}=s_{0,ij}.(1-x_{ik})+{}_{ik}\;(10)$$

where u _ijk_ is the observation k of the mixture effect on the basal area increment of species i when mixed with species j and for a proportion of species i of x _ik_; s _0_ is a parameter to be estimated, representing the effect of species j on the basal area increment of species i, and is the residual error term. In this equation, the effect of mixture (u) tends towards 0 when the proportion of the species under consideration (x) tends towards 1 ( *i.e.* when tending towards a pure stand). s _0_ > 0 indicates a positive effect of mixture on the growth of species i while s _0_ < 0 indicates a negative effect.

Several authors identified a variation of the mixture effect with site productivity ( Paquette & Messier, 2011; Toïgo *et al*., 2015). To account for this, we first calculated the species-specific site indices (SI) for every mixed stand inventory plots using Equation 2 and the parameter estimates shown in Table 4. We then added the site indices to the mixture effect model (described in Equation 10) as follows:

$$\;u_{ijk}=(s_{0,ij}+s_{1,ij}.SI_{ik}).(1-x_{ik})+{}_{ik}\;(11)$$

where s _0_ and s _1_ are parameters to be estimated. s _1_ > 0 indicates the mixture effect on species productivity increases with site productivity. Conversely, s _1_ < 0, indicates the mixture effect decreases with site productivity. We fitted the models presented at Equation 10 and Equation 11 with the *gnls* function from the *nlme R* package ( Pinheiro *et al*., 2020).

Salem uses either of these models to simulate species growth in mixed stands. If site productivity has a significant effect on the mixture effect, then Equation 11 is used to simulate the growth of the species under consideration. Conversely, if site productivity has a non-significant effect on the mixture effect, Salem only considers the average mixture effect and Equation 10 is used. Table 6 presents the parameter estimates of Equation 10 and Equation 11 used in Salem. All parameter estimates of Equation 10 and Equation 11 are presented in Tables A.1 and A.2 of the extended data ( Aussenac *et al*., 2021).

**Table 6.** Estimates of the s _0_ ans s _1_ parameters in Equation 10 and Equations 11 used in Salem. ns.: non-significant. For a given species pair, if s _1_ is non-significant, then the s _0_ estimate presented comes from Equation 10. On the other hand, if s _1_ is significant, then both s _0_ and s _1_ estimates come from Equation 11. Correspondence between species full names and their abbreviations is presented in Table 2.

Effect of													
		*param.*	*Qu. ro.*	*Qu. pe.*	*Qu. pu.*	*Fa. sy.*	*Pi. pi.*	*Pi. sy.*	*Pi. la.*	*Pi. ni.*	*Ab. al.*	*Pi. ab.*	*Ps. me.*
On the growth of	*Qu. ro.*	s _0_		ns.	ns.	ns.	0.692	0.374				ns.	ns.
		s _1_		ns.	ns.	ns.	ns.	ns.				ns.	ns.
	*Qu. pe.*	s _0_	0.215			0.618		0.253			ns.		ns.
		s _1_	ns.			-0.022		ns.			ns.		ns.
	*Qu. pu.*	s _0_	2.133					ns.					
		s _1_	-0.323					ns.					
	*Fa. sy.*	s _0_	2.299	0.833				0.714			0.544	0.435	
		s _1_	-0.058	-0.019				ns.			ns.	ns.	
	*Pi. pi.*	s _0_	0.384						ns.				
		s _1_	ns.						ns.				
	*Pi. sy.*	s _0_	ns.	ns.	0.902	0.110			1.025	ns.	-0.406	ns.	ns.
		s _1_	ns.	ns.	ns.	-0.012			ns.	ns.	ns.	ns.	ns.
	*Pi. la.*	s _0_					ns.	ns.					
		s _1_					ns.	ns.					
	*Pi. ni.*	s _0_						1.419					
		s _0_						-0.017					
	*Ab. al.*	s _0_		2.080		0.996		0.991				0.910	2.371
		s _1_		-0.060		-0.032		ns.				-0.024	-0.060
	*Pi. ab.*	s _0_	ns.			ns.		0.417			1.792		1.176
		s _1_	ns.			ns.		ns.			-0.024		-0.016
	*Ps. me.*	s _0_	ns.	ns.				ns.			0.259	ns.	
		s _1_	ns.	ns.				ns.			ns.	ns.	

In Salem, species growth in mixed stands is therefore calculated by multiplying species growth in pure stands (BAI as defined in Equation 1) by the species proportion in the stand (x as defined in Equation 8) and by the mixture effect (u as defined in Equation 10 and Equation 11). Thus, if site productivity has a significant effect on the mixture effect, then species growth in mixed stand is calculated as follows:

$$BAI_{ijk}=SI_{ik}\;.\;f_{2,i}(DI_{k})\;.\;f_{3,i}(Dg_{ik}).x_{ik}.(1+(s_{0,ij}+s_{1,ij}.SI_{ik}).(1-x_{ik}))+{}_{ijk}\;(12)$$

On the other hand, if site productivity has a non-significant effect on the mixture effect, then species growth in mixed stand is calculated as follows:

$$BAI_{ijk}=SI_{ik}\;.\;f_{2,i}(DI_{k})\;.\;f_{3,i}(Dg_{ik}).x_{ik}.(1+(s_{0,ij}).(1-x_{ik}))+{}_{ijk}\;(13)$$

**Figure 2.**
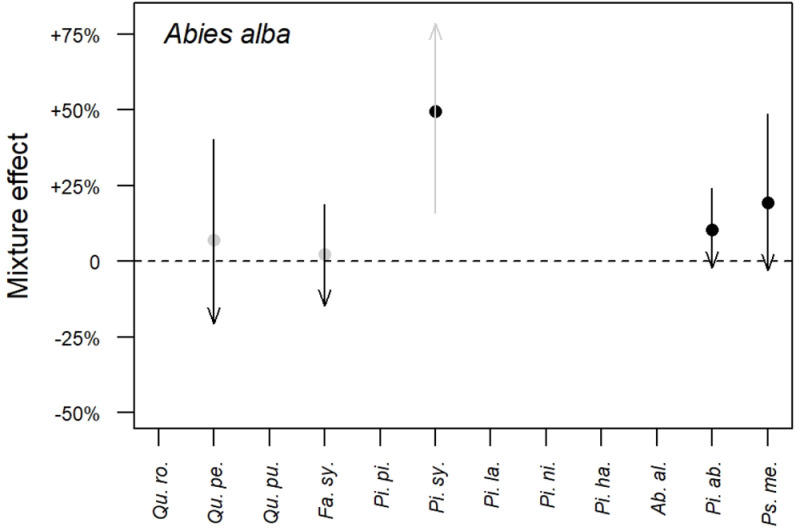
Mixture effect on fir ( *Abies alba*) growth. Dots indicate the average mixture effect calculated at Equation 10. Arrows indicate the variation of the mixture effect with site productivity calculated at Equation 11: the beginning of the arrows represents the poorest sites (5% quantile) while the tip of the arrows represents the richest sites (95% quantile). A grey colour indicates that the effect is non-significant, while a black colour indicates a significant effect. Correspondence between species full names and their abbreviations is presented in Table 2.

***3.2.3 Results synthesis*.** As an example, Figure 2 synthesises the results obtained on the mixture effect on fir ( *Abies alba*) growth. At the poorest sites ( *i.e.* at sites with lowest SI) *Quercus petraea* and *Fagus sylvatica* tend to increase fir growth while they tend to decrease its growth at the richest sites ( *i.e.* at sites with highest SI). At the poorest sites, *Picea abies* and *Pseudotsuga menziesii* also tend to increase fir growth but as sites become richer, this effect tends towards zero. For those two species a positive average mixture effect is also present. As for *Pinus sylvestris*, it increases fir growth by about 50% whatever the site richness, the effect of site richness being non-significant. The mixture effect on the other species is presented in Figure A.1 of the extended data ( Aussenac *et al*., 2021).

We found the mixture effect on species growth was most often positive ( *i.e.* s _0_>0 in Equation 10) when significant. Only *F. sylvatica* and *A. alba* had a negative effect on *P. sylvestris* growth. In addition, we found the effect of site productivity on the mixture effect was always negative (s _1_<0 in Equation 11) when significant. This indicates the effect of mixture is always stronger at poor sites than at richer sites, consistent with the stress gradient hypothesis ( Bertness & Callaway, 1994). These results complement those presented in Toïgo *et al*. (2015) for five species pairs.

### 3.3 Models of bark proportion

***3.3.1 Data*.** We calibrated our bark models using measurements of bark proportion (expressed as a proportion of the trees BA) collected by the French NFI service. We had at our disposal, for nine species or groups of species and for four to five dbh classes: the number of sampled trees, their mean dbh and the mean and variance of their bark proportion. All data can be found in Bouvet & Deleuze (2013). In this dataset, *Q. robur* and *Q. petraea* are not differentiated and no data are available for *P. halepensis*. We therefore built a single bark model for both *Quercus* species and Salem applies the *P. sylvestris* bark model to *P. halepensis* because these two species have a very similar bark proportion (Deleuze, personal communications).

**Figure 3.**
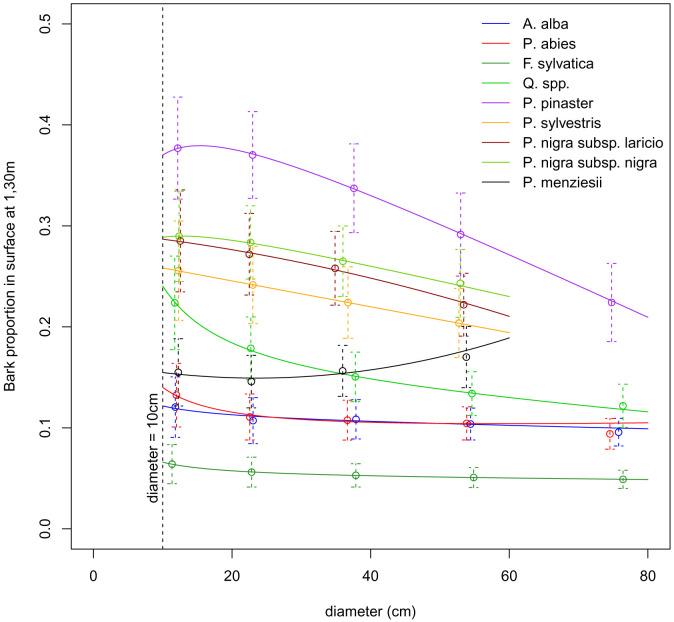
Prediction of bark proportion as a function of diameter at breast heigh (dbh). Dots show the mean bark proportion for each species or group of species and each dbh class. Dashed vertical lines show the dispersion of the bark proportion measures. Solid lines show the models predictions.

***3.3.2 Models*.** We built three different models to describe the relationship between bark proportion (BP) and dbh. Each species was assigned the model that best fitted its data, *i.e.* the model with the lower AIC value ( Table 7). We calibrated the models by maximising likelihood, taking into account the error of each bark proportion value. We calculated this error using the number of sampled trees and the variance associated to each bark proportion value. Models were fitted with the *mle2* function from the *bbmle R* package. The models parameter estimates are presented in Table 7 and the models predictions are presented in Figure 3. For tree diameters below 10 cm, or above the maximum dbh value indicated in Table 7, Salem uses the predicted bark proportion at 10 cm or at the maximum dbh value in order to avoid using the models outside their calibration range. We defined the minimum dbh value of 10 cm and the maximum dbh values of 60 or 80 cm by rounding the species minimum value of dbh down to the nearest ten and their maximum value of dbh to the upper ten.

**Table 7.** Bark models and their parameter estimates for each species or group of species. Standard errors are shown in parentheses. Levels of significance: *p<0.05, **p<0.01, ***p<0.001. Correspondence between species full names and their abbreviations is presented in Table 2.

Species	Model	a	b	c	Max dbh
*Quercus spp.*	*a* + *b* * *dbh* + *c*/ *dbh*	0.1426 *** (0.0011)	-0.000496 *** (0.000018)	1.028 *** (0.013)	80
*Fa. sy.*	*a* + *b* * *dbh* + *c*/ *dbh*	0.0502 *** (0.0007)	-0.000042 *** (0.000012)	0.162 *** (0.008)	80
*Pi. pi.*	*a* + *b* * *dbh* + *c*/ *dbh*	0.4792 *** (0.004)	-0.003252 *** (0.000074)	-0.765 *** (0.043)	80
*Pi. sy.*	*a* + *b* * *dbh*	0.2711 *** (0.001)	-0.00128 *** (0.000032)		60
*Pi. la.*	*a* + *b* * *dbh* + *c* * *dbh* ^2^	0.2948 *** (0.0045)	-0.000679 * (0.000344)	-1.21e-5 * (0.59e-5)	60
*Pi. ni.*	*a* + *b* * *dbh* + *c*/ *dbh*	0.3311 *** (0.0079)	-0.001611 *** (0.000161)	-0.263 *** (0.082)	60
*Ab. al.*	*a* + *b* * *dbh* + *c*/ *dbh*	0.1084 *** (0.0016)	-0.000139 *** (0.000025)	0.148 *** (0.019)	80
*Pi. ab.*	*a* + *b* * *dbh* + *c*/ *dbh*	0.0864 *** (0.0015)	0.000151 *** (0.000027)	0.522 *** (0.016)	80
*Ps. me.*	*a* + *b* * *dbh* + *c*/ *dbh*	0.1659 *** (0.0018)	-0.001409 *** (0.000136)	3.0e-5 *** (0.2e-5)	60

### 3.4 Mortality

Mortality is triggered when stand density exceeds the self-thinning boundary, *i.e.* when DI (in Equation 6 or Equation 9) is greater than or equal to 1. In that case, DI is reduced to 1 using the Kg parameter, with

$$\;Kg=Dg_{d}^{2}/Dg^{2}=0.68\;(14)$$

where Dg _d_ is the mean quadratic diameter of killed trees and Dg is the mean quadratic diameter of trees before mortality is applied. This value of Kg = 0.68 is based on an analysis of the French NFI mortality data.

To reduce DI to 1, the number of trees in the stand is reduced while the mean quadratic diameter is increased, which amounts to preferentially killing small trees. In mixed stands, the number of trees killed for each species is defined according to their proportion in the stand before mortality process.

In practice in Salem, the number of trees and the mean quadratic diameter after mortality are found using a system of two equations: Equation 6, which links DI, Dg and N, and Equation 14, which quantifies the size of the trees to be killed, while knowing that DI after mortality equals 1. This system cannot be solved analytically; we use a dichotomy algorithm to obtain the post-mortality values.

### 3.5 Models of individual tree diameter distribution

***3.5.1 Data*.** We worked with the same NFI plots as those used to calibrate our growth models, *i.e.* with NFI data collected between 2006 and 2013 in pure stands ( Table 2). We used the tree diameter measurements to calibrate our distribution models.

**Figure 4.**
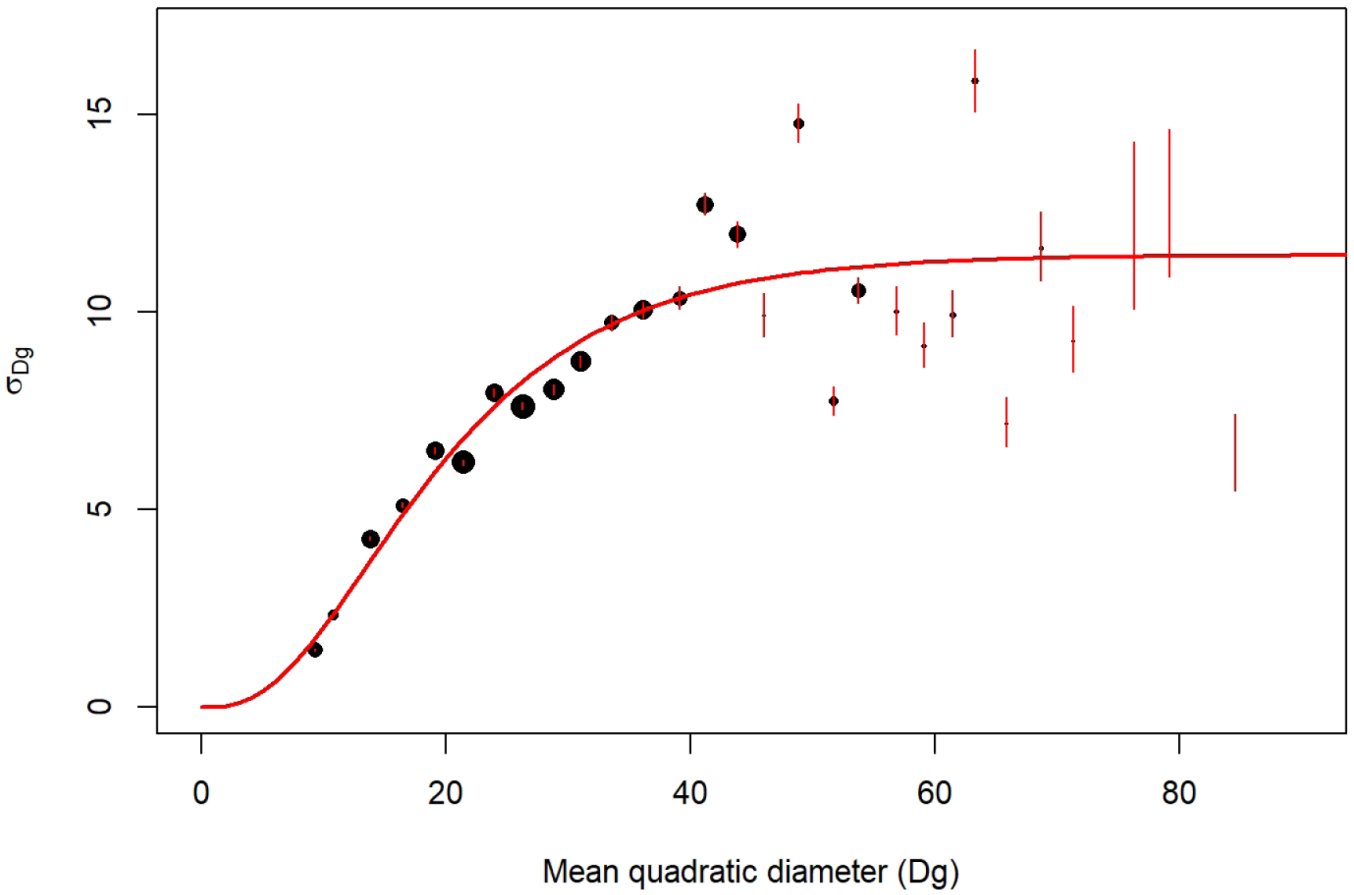
*σ* parameter variation with mean quadratic diameter (Dg). Example for *Quercus robur*. Point size is proportional to the number of NFI plots. The red segments are the confidence interval of σ. The red line corresponds to the predictions of σ modelled with Equation 15.

***3.5.2 Models*.** We built species-specific models predicting the diameter distribution of individual trees from their mean quadratic diameter and their total number in the stands. For that, we followed a three-step procedure: first, we grouped the NFI plots by Dg classes. Second, within each class we fitted a normal distribution to the observed distribution of individual tree diameter, *i.e.* the number of stems per diameter value (Figure A.2 of the extended data ( Aussenac *et al*., 2021)). Each fit provides a standard deviation ( *σ*) for the normal distribution. Third, we modelled the obtained standard deviations as a function of the mean quadratic diameter ( Figure 4). For that we used the following sigmoidal equation (Chapman-Richards type model; Pienaar & Turnbull, 1973):

$$\;\sigma_{ik}=\alpha_{i}.{\left(1-e^{\left(-\gamma_{i}*Dg_{ik}\right)})\right)}^{\beta_{i}}+{}_{ik}\;(15)$$

where *σ*_ik_ corresponds to the observation k of the standard deviations of the normal distributions fitted at the second step for species i; Dg corresponds to the mean quadratic diameter of all plots within each diameter class; *α*, *β*, and *γ* are three parameters to be estimated and is the residual error term. The parameter estimates are presented in Table 8.

**Table 8.** Species-specific parameter estimates for the models of individual tree diameter distribution. Levels of significance: * p<0.05; ** p<0.01; *** p<0.001; ns.: non-significant. Correspondence between species full names and their abbreviations is presented in Table 2.

Species	Parameter	Estimate	Std. error	Significance
*Qu. ro.*	*α*	11.43876	0.72894	***
	*γ*	0.09017	0.02740	**
	*β*	3.33197	1.71724	ns.
*Qu. pe.*	*α*	10.97643	0.58223	***
	*γ*	0.08640	0.01970	***
	*β*	3.22690	1.17333	*
*Qu. pu.*	*α*	7.72100	0.39835	***
	*γ*	0.18849	0.04261	**
	*β*	8.74034	5.05425	ns.
*Fa. sy.*	*α*	12.24093	0.65589	***
	*γ*	0.07743	0.01841	***
	*β*	2.85483	1.04041	**
*Pi. pi.*	*α*	6.65825	0.23426	***
	*γ*	0.08697	0.01528	***
	*β*	2.14814	0.52160	***
*Pi. sy.*	*α*	8.56996	0.90385	***
	*γ*	0.08513	0.03516	*
	*β*	2.10282	1.07330	ns.
*Pi. la.*	*α*	7.57343	1.01858	***
	*γ*	0.07817	0.02498	*
	*β*	2.57583	0.84827	*
*Pi. ni.*	*α*	6.35432	0.45823	***
	*γ*	0.16440	0.05525	*
	*β*	5.72509	4.07794	ns.
*Pi. ha.*	*α*	8.95631	0.50538	***
	*γ*	0.18881	0.05684	**
	*β*	9.68719	7.70238	ns.
*Ab. al.*	*α*	13.25233	1.49189	***
	*γ*	0.05094	0.02006	*
	*β*	1.76612	0.73376	*
*Pi. ab.*	*α*	30.18095	62.23563	ns.
	*γ*	0.00754	0.02397	ns.
	*β*	0.94366	0.38691	*
*Ps. me.*	*α*	7.38019	0.32657	***
	*γ*	0.12265	0.02533	***
	*β*	4.59548	1.80814	*

In practice in Salem, to generate the diameter distribution of a stand we calculate the number of trees for each diameter class within the stand using the following equation:

$$\;N_{c}=N_{tot}.\;W_{c}.\frac{1}{\sigma .\sqrt{2\pi }}\;.\;e^{-\frac{1}{2}\frac{{\left(dbh_{c}\;-\;Dm\right)}^{2}}{\sigma^{2}}}\;(16)$$

where N _c_ is the number of trees of diameter class c, N _tot_ is the total number of trees within the stand, W _c_ is the c class width, *σ* is predicted from Equation 15, dbh _c_ is the central dbh value of diameter class c and Dm is the arithmetic mean diameter of the diameter distribution. Thanks to the properties of the normal distribution, the arithmetic mean Dm and the quadratic mean Dg are linked by the following equation:

$$\;Dm=\sqrt{Dg^{2}-\sigma^{2}}\;(17)$$

### 3.6 Models of individual tree height

***3.6.1 Data*.** We calibrated our height-circumference relationship models using the same data used to calibrate our growth models, *i.e.* French NFI data collected between 2006 and 2013 in pure stands. However, here we excluded trees with defects: broken trees and pollards. Differences in the number of plots used to calibrate our growth models and our height-circumference relationship models is due to the fact that (1) here we removed a few plots consisting solely of broken trees and/or pollards and (2) here we considered pure stands excluded for growth modelling: those with missing environmental variables and *P. pinaster* stands with a quadratic diameter smaller than 5 cm. The number of plots and trees per species are presented in Table 9.

**Table 9.** Number and features of inventory plots in pure stands used for the height-circumference relationship models for each species. Plots nb is the number of NFI plots used for the models calibration; Nb stems is the total number of trees at the plots; c _130_ corresponds to the tree circumferences in cm and h _tot_ to the tree heights in m. Correspondence between species full names and their abbreviations is presented in Table 2.

	Plots nb	Nb stems total	c _130_		h _tot_	
Species			min	max	min	max
*Qu. ro.*	487	2903	24	338	3.9	37.8
*Qu. pe.*	612	4310	24	370	3.7	43.1
*Qu. pu.*	229	1303	24	314	3.0	30.0
*Fa. sy.*	554	4412	24	427	3.0	45.0
*Pi. pi.*	1276	8597	24	349	3.5	38.4
*Pi. sy.*	613	4797	24	259	2.5	33.4
*Pi. la.*	224	1725	24	362	3.1	36.5
*Pi. ni.*	156	1167	24	200	3.2	28.7
*Pi. ha.*	165	1153	24	249	3.4	24.8
*Ab. al.*	261	2782	24	345	4.4	42.7
*Pi. ab.*	525	5019	24	347	3.8	42.8
*Ps. me.*	542	4646	24	246	4.3	43.6

***3.6.2 Models*.** We modelled tree height from tree circumference while taking into account the effects of stand development stage and site productivity. Our models are given by the following generic equation:

$$h_{tot,ik}=1.3+(\alpha_{i}+g_{1,i}(Dg_{ik})+g_{2,i}(SI_{ik}))\left(1-e^{-\beta_{i}c_{130,ik}^{\gamma_{i}}}\right)+{}_{ik}\;(18)$$

where h _tot,ik_ and c _130,ik_ are the total height and circumference at 1.30 m of tree k of species i; Dg is the stand mean quadratic diameter used as a proxy of the stand development stage; SI is the site index as described in Equation 2; *α*, *β* and *γ* are parameters to be estimated and is the residual error term. Functions g _1_ and g _2_ could be either linear ( *a*. *x*), quadratic ( *a.x* + *b.x* ^2^), exponential towards an asymptote ( *a*(1 − *e* ^− *b.x*^)) or logarithmic ( *a.ln*( *x*)).

**Figure 5.**
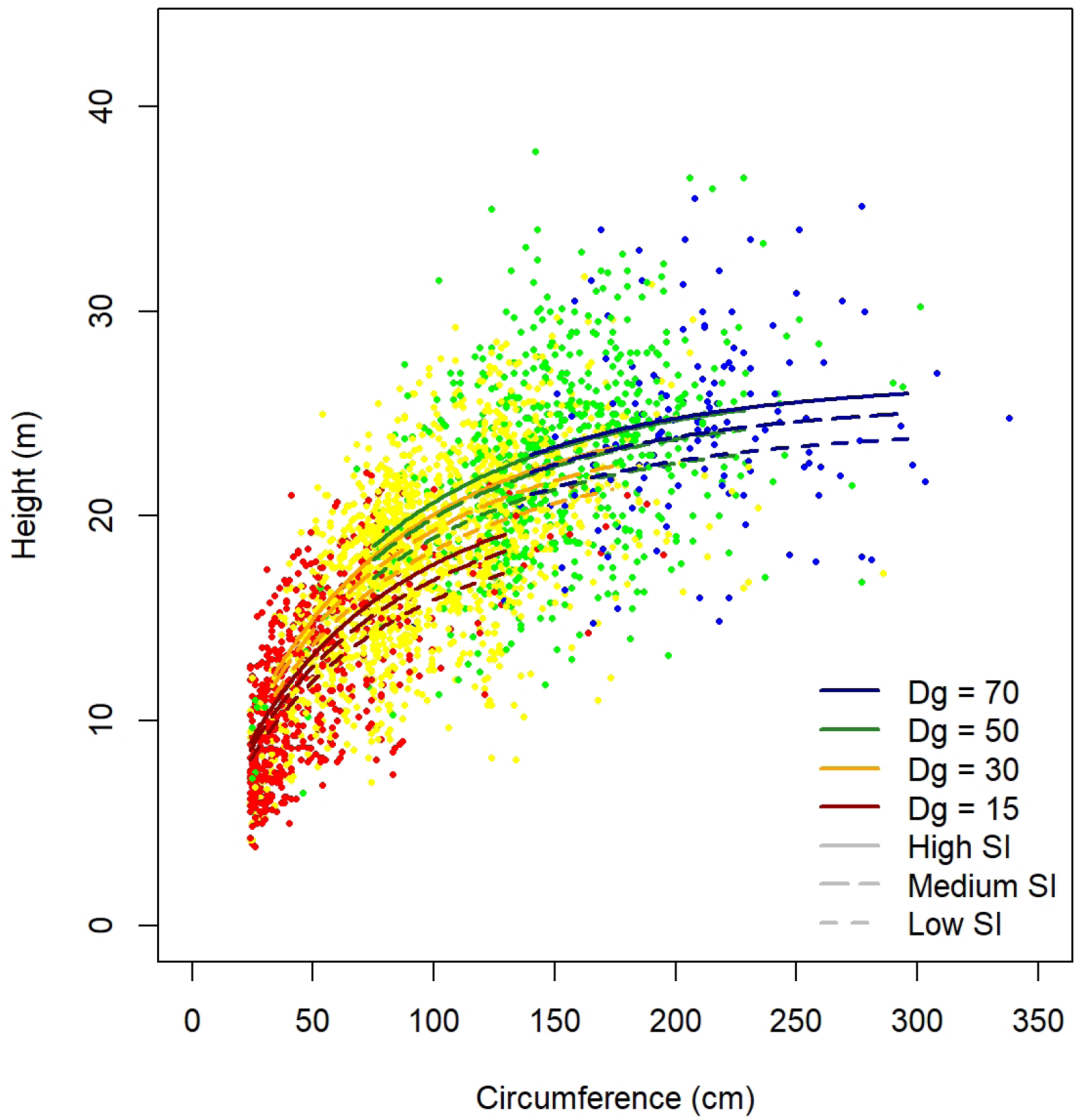
Height predictions for *Quercus robur* as a function of tree circumference, stand quadratic diameter (Dg) and site index (SI).

For each species, we selected the combination of g _1_ and g _2_ functions giving the lowest AIC value. When the difference between models was less than 4 AIC points, monotonic functions were chosen in preference to polynomial functions to avoid possible prediction errors outside of the calibration range. The selected models are presented in Table 10 and their associated parameter estimates are presented in Table 11. As an example, height predictions for *Quercus robur* are presented in Figure 5.

**Table 10.** Height-circumference models used for each species. Correspondence between species full names and their abbreviations is presented in Table 2.

Species	Models
*Qu. ro.*	( *α* + *a*_1.1_(1 – *e* ^– *a*_1.2_ *Dg*^) + *a*_2_ *SI*)(1 – $e^{-\beta c_{130}^{\gamma }}$ ) + 1.3
*Qu. pe.*	( *α* + *a*_1_ *Dg* + *a*_2.1_ *SI* + *a*_2.2_ *SI* ^2^)(1 – $e^{-\beta c_{130}^{\gamma }}$ ) + 1.3
*Qu. pu.*	( *α* + *a*_2.1_ *SI* + *a*_2.2_ *SI* ^2^)(1 – $e^{-\beta c_{130}^{\gamma }}$ ) + 1.3
*Fa. sy.*	( *α* + *a*_1.1_(1 – *e* ^– *a*_1.2_ *Dg*^) + *a*_2.1_ *SI* + *a*_2.2_ *SI* ^2^)(1 – $e^{-\beta c_{130}^{\gamma }}$ ) + 1.3
*Pi. pi.*	( *α* + *a*_1.1_ *Dg* + *a*_1.2_ *Dg* ^2^ + *a*_2.1_ *SI* + *a*_2.2_ *SI* ^2^)(1 – $e^{-\beta c_{130}^{\gamma }}$ ) + 1.3
*Pi. sy.*	( *α* + *a*_1.1_(1 – *e* ^– *a*_1.2_ *Dg*^) + *a*_2_ *SI*)(1 – $e^{-\beta c_{130}^{\gamma }}$ ) + 1.3
*Pi. la.*	( *α* + *a*_1.1_(1 – *e* ^– *a*_1.2_ *Dg*^) + *a*_2.1_ *SI* + *a*_2.2_ *SI* ^2^)(1 – $e^{-\beta c_{130}^{\gamma }}$ ) + 1.3
*Pi. ni.*	( *α* + *a*_1.1_(1 – *e* ^– *a*_1.2_ *Dg*^))(1 – $e^{-\beta c_{130}^{\gamma }}$ ) + 1.3
*Pi. ha.*	( *α* + *a*_1.1_(1 – *e* ^– *a*_1.2_ *Dg*^) + *a*_2_ *log(SI)*)(1 – $e^{-\beta c_{130}^{\gamma }}$ ) + 1.3
*Ab. al.*	( *α* + *a*_1.1_(1 – *e* ^– *a*_1.2_ *Dg*^) + *a*_2_ *log(SI)*)(1 – $e^{-\beta c_{130}^{\gamma }}$ ) + 1.3
*Pi. ab.*	( *α* + *a*_1.1_(1 – *e* ^– *a*_1.2_ *Dg*^) + *a*_2.1_(1 – *e* ^– *a*_2.2_ *SI*^))(1 – $e^{-\beta c_{130}^{\gamma }}$ ) + 1.3
*Ps. me.*	( *α* + *a*_1.1_(1 – *e* ^– *a*_1.2_ *Dg*^)(1 – $e^{-\beta c_{130}^{\gamma }}$ ) + 1.3

**Table 11.** Parameter estimates of the height-circumference models for each species. Standard errors are shown in parentheses. Levels of significance: * p<0.1; ** p<0.05; *** p<0.01; ns.: non-significant. Correspondence between species full names and their abbreviations is presented in Table 2.

	*Qu. ro.*	*Qu. pe.*	*Qu. pu.*	*Fa. sy.*	*Pi. pi.*	*Pi. sy.*	*Pi. la.*	*Pi. ni.*	*Pi. ha.*	*Ab. al.*	*Pi. ab.*	*Ps. me.*
*α*	-1.3624ns (4.0668)	2.1484 * (1.2733)	-3.7295 *** (1.4019)	-5.8023 *** (1.9697)	-5.1515 *** (0.7774)	-4.4002 ** (1.8007)	-20.9182 *** (4.5868)	-6.342 * (3.244)	-26.1684 *** (8.0869)	0.8475 * (2.4901)	-30.0783 *** 8.4595	-5.5879 *** (1.7884)
a _1_		0.1692 *** (0.0065)										
a _1.1_	17.9437 *** (4.0668)			15.8848 *** (1.5124)	0.6972 *** (0.026)	16.303 *** (1.4992)	45.475 *** (5.212)	29.5273 *** (3.5529)	24.4999 *** (7.0713)	23.0394 *** (1.6357)	45.4929 *** 3.4257	50.3923 *** (2.8952)
a _1.2_	0.099 *** (0.0159)			0.0491 *** (0.0071)	-0.0058 *** (0.0004)	0.0656 *** (0.0097)	0.0645 *** (0.0059)	0.0725 *** (0.0101)	0.1001 *** (0.0164)	0.0411 *** (0.0059)	0.0897 *** 0.0052	0.0487 *** (0.0027)
a _2_	0.4222 *** (0.0336)					0.1541 *** (0.0049)			7.659 *** (1.7059)	3.1832 *** (0.3481)		
a _2.1_		0.8495 *** (0.0815)	5.4023 *** (0.4581)	1.1844 *** (0.065)	0.3408 *** (0.0246)		0.3808 *** (0.095)				17.3624 ** 7.4193	
a _2.2_		-0.0114 *** (0.0013)	-0.3118 *** (0.0341)	-0.0145 *** (0.0011)	-0.0026 *** (0.0002)		-0.0037 *** (0.0008)				0.0497 *** 0.0127	
*β*	0.0314 *** (0.005)	0.0067 *** (0.0008)	0.0102 *** (0.0026)	0.0137 *** (0.0016)	0.0238 *** (0.0025)	0.0168 *** (0.003)	0.0348 *** (0.007)	0.0224 *** (0.0069)	0.0689 *** (0.0131)	0.0075 *** (0.0009)	0.016 *** 0.0019	0.0355 *** (0.0046)
*γ*	0.8341 *** (0.046)	1.2896 *** (0.0312)	1.1533 *** (0.0727)	1.0219 *** (0.0313)	0.9357 *** (0.0288)	1.0502 *** (0.0519)	0.7536 *** (0.0701)	0.9475 *** (0.0958)	0.6054 *** (0.0968)	1.1343 *** (0.0328)	0.9984 *** 0.0337	0.7609 *** (0.0441)

### 3.7 Models of individual tree volume

***3.7.1 Data*.** The models of individual tree volume were fitted to a large dataset that was mainly collected by the French National Institute of Agricultural Research between 1920 and 1983. During that period, a large sample of harvested trees was selected and the volume of each individual tree was measured from the base up to a 7 cm small-end diameter ( Deleuze *et al*., 2013). This large dataset was later complemented by two field campaigns in 2009 and 2010. Before screening, it included 44,173 volume measurements of individual trees. Trees that did not reach the French commercial limit (dbh = 7.5 cm ) were discarded.

The dataset originally included 52 species, but some of them were scarce and therefore we focused on those for which we had at least 30 volume observations. After discarding the scarce species, the dataset that was used to fit the species-specific models contained 42,959 observations shared among 30 species. The characteristics of the dataset for the species relevant to Salem are shown in Table 12. The information on the other species can be found in Table A.3 of the extended data ( Aussenac *et al*., 2021).

**Table 12.** Mean diameter at breast heigh (dbh), height and volume of trees in the volume dataset, for species that are relevant to Salem. Minimum and maximum values are shown in parentheses. *^a^* Number of observations. Correspondence between species full names and their abbreviations is presented in Table 2. *^b^* Undistinguished *Quercus sp.* used for *Quercus pubescens*.

Species	n *^a^*	dbh (cm)	height (m)	volume (dm ^3^)
*Qu. ro.*	84	15.6 (7.6, 27.7)	14.6 (6.0, 20.8)	174 (17, 711)
*Qu. pe.*	8018	20.9 (7.6, 146.7)	19.8 (2.0, 42.0)	604 (4, 15991)
*Qu. sp. ^b^*	1416	23.2 (7.6, 101.9)	18.2 (7.0, 42.5)	715 (11, 10114)
*Fa. sy.*	7099	24.2 (7.6, 108.5)	22.2 (3.0, 44.0)	776 (6, 16234)
*Pi. pi.*	2881	19.8 (7.6, 66.5)	11.6 (4.5, 27.0)	218 (5, 4125)
*Pi. sy.*	5216	23.7 (7.6, 81.8)	19.3 (4.0, 31.2)	552 (6, 7862)
*Pi. la.*	1163	28.3 (8.0, 70.3)	22.4 (8.9, 36.4)	907 (18, 5908)
*Pi. ni.*	847	19.2 (7.6, 47.7)	15.6 (6.2, 27.9)	294 (7, 1739)
*Pi. ha.*	257	24.8 (7.6, 64.0)	14.6 (7.8, 21.5)	401 (13, 2315)
*Ab. al.*	5835	32.9 (7.6, 103.0)	23.7 (4.0, 41.0)	1528 (14, 11878)
*Pi. ab.*	2715	23.9 (7.6, 79.9)	21.5 (5.5, 43.5)	803 (7, 9215)
*Ps. me.*	2355	18.6 (7.6, 57.3)	17.7 (5.4, 41.5)	365 (7, 4425)

***3.7.2 Models*.** We modelled tree volume from their dbh and total height. We built species-specific models to account for the differences in tree form across species. These models were given by:

$$\;\upsilon_{ik}=\beta_{1,i}\frac{h_{tot,ik}}{dbh_{ik}}+(\beta_{2,i}+\beta_{3,i}dbh_{ik})\frac{\pi dbh_{ik}^{2}h_{tot,ik}}{40}+{}_{ik}\;(19)$$

where v _ik_ is the commercial volume (dm ^3^) of tree k of species i, that is the over bark volume of the bole from the base to a small-end diameter of 7 cm excluding the branches; h _tot_ is the tree total height (m); dbh is the tree diameter (cm); *β*_1_, *β*_2_, and *β*_3_ are parameters to be estimated and is the residual error term. This error term is assumed to be normally distributed with mean 0 and variance $dbh_{ik}^{4}\sigma_{i}^{2}$ . Term $\pi dbh_{ik}^{2}h_{tot,ik}$ /40 represents the volume of a cylinder and the factor 40 ensures a proper unit conversion. Parameter *β*_2_ is considered as a form factor, that is the ratio between the volume of the tree and that of the cylinder ( Pretzsch, 2009, p. 183). Parameter *β*_3_ represents an adjustment to this form factor that increases or decreases with the diameter. Parameter *β*_1_ is related to the non commercial part of the the tree, that is the part beyond the 7-cm small-end diameter. Its value is expected to be negative.

The model shown in Equation 19 was fitted to each species. Whenever an effect was found to be non-significant, it was removed from the model. The parameter estimates for the species that are relevant to Salem are presented in Table 13. For the other species, the reader is referred to Table A.4 of the extended data ( Aussenac *et al*., 2021).

**Table 13.** Species-specific parameter estimates of the volume models ( Equation 19), for species that are relevant to Salem. Standard errors are shown in parentheses. All the parameter estimates are highly significant (p < 0.001). Correspondence between species full names and their abbreviations is presented in Table 2. ^a^Undistinguished *Quercus sp.* used for *Quercus pubescens*.

Species	*β*_1_	*β*_2_	*β*_3_	*σ* ^2^
*Qu. ro.*		5.057×10 ^–1^ (0.074×10 ^–1^)		6.355×10 ^–3^
*Qu. pe.*	-4.438 (0.193)	5.455×10 ^–1^ (0.016×10 ^–1^)	-1.397×10 ^–3^ (0.041×10 ^–3^)	6.835×10 ^–3^
*Qu. sp. ^a^*	-3.319 (0.387)	5.664×10 ^–1^ (0.040×10 ^–1^)	-1.778×10 ^–3^ (0.102×10 ^–3^)	6.094×10 ^–3^
*Fa. sy.*	-4.498 (0.195)	5.107×10 ^–1^ (0.017×10 ^–1^)	-1.332×10 ^–3^ (0.045×10 ^–3^)	8.037×10 ^–3^
*Pi. pi.*	-1.712 (0.430)	5.130×10 ^–1^ (0.051×10 ^–1^)	-1.124×10 ^–3^ (0.196×10 ^–3^)	2.363×10 ^–3^
*Pi. sy.*	-2.247 (0.271)	4.985×10 ^–1^ (0.027×10 ^–1^)	-1.212×10 ^–3^ (0.081×10 ^–3^)	6.348×10 ^–3^
*Pi. la.*		5.215×10 ^–1^ (0.045×10 ^–1^)	-1.271×10 ^–3^ (0.129×10 ^–3^)	8.547×10 ^–3^
*Pi. ni.*	-8.427 (0.768)	5.857×10 ^–1^ (0.093×10 ^–1^)	-2.748×10 ^–3^ (0.349×10 ^–3^)	4.618×10 ^–3^
*Pi. ha.*		5.258×10 ^–1^ (0.091×10 ^–1^)	-2.300×10 ^–3^ (0.314×10 ^–3^)	3.110×10 ^–3^
*Ab. al.*	-4.523 (0.494)	6.011×10 ^–1^ (0.019×10 ^–1^)	-2.243×10 ^–3^ (0.039×10 ^–3^)	7.116×10 ^–3^
*Pi. ab.*	-8.666 (0.278)	5.839×10 ^–1^ (0.026×10 ^–1^)	-2.679×10 ^–3^ (0.066×10 ^–3^)	5.747×10 ^–3^
*Ps. me.*	-7.150 (0.316)	5.431×10 ^–1^ (0.038×10 ^–1^)	-2.951×10 ^–3^ (0.121×10 ^–3^)	4.493×10 ^–3^

## 4 Operation

Salem runs on the Capsis open software framework ( Dufour-Kowalski *et al*., 2012) which ensures its availability and code continuity and facilitates interactions with other forest models and simulators. It is distributed under a LGPL license and is therefore free and open source. As the underlying code of Capsis and Salem is in Java, simulations can be performed with any operating system (Windows, Linux, or Mac). Finally, Salem’s clear graphical user interface makes it easy to use for non-modellers.

### 4.1 Stand initialisation

**Figure 6.**
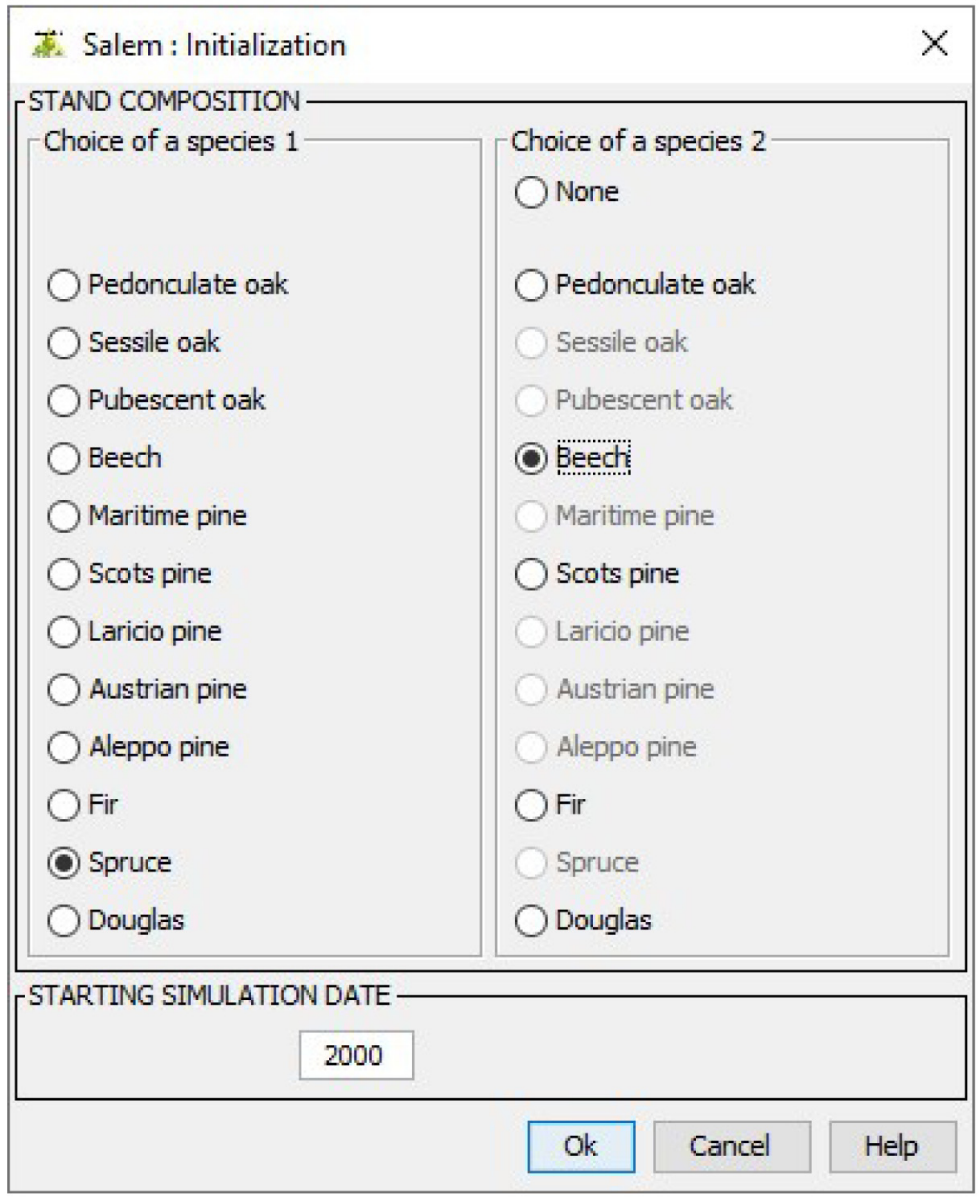
Choosing a composition and a starting simulation date.

After choosing Salem from the menu showing the different models available in Capsis, users must first specify the composition of the stand to simulate and a starting simulation date. For this demonstration, we choose to simulate a stand made up of spruce ( *Picea abies*) and beech ( *Fagus sylvatica*) from 2000 onwards ( Figure 6).

**Figure 7.**
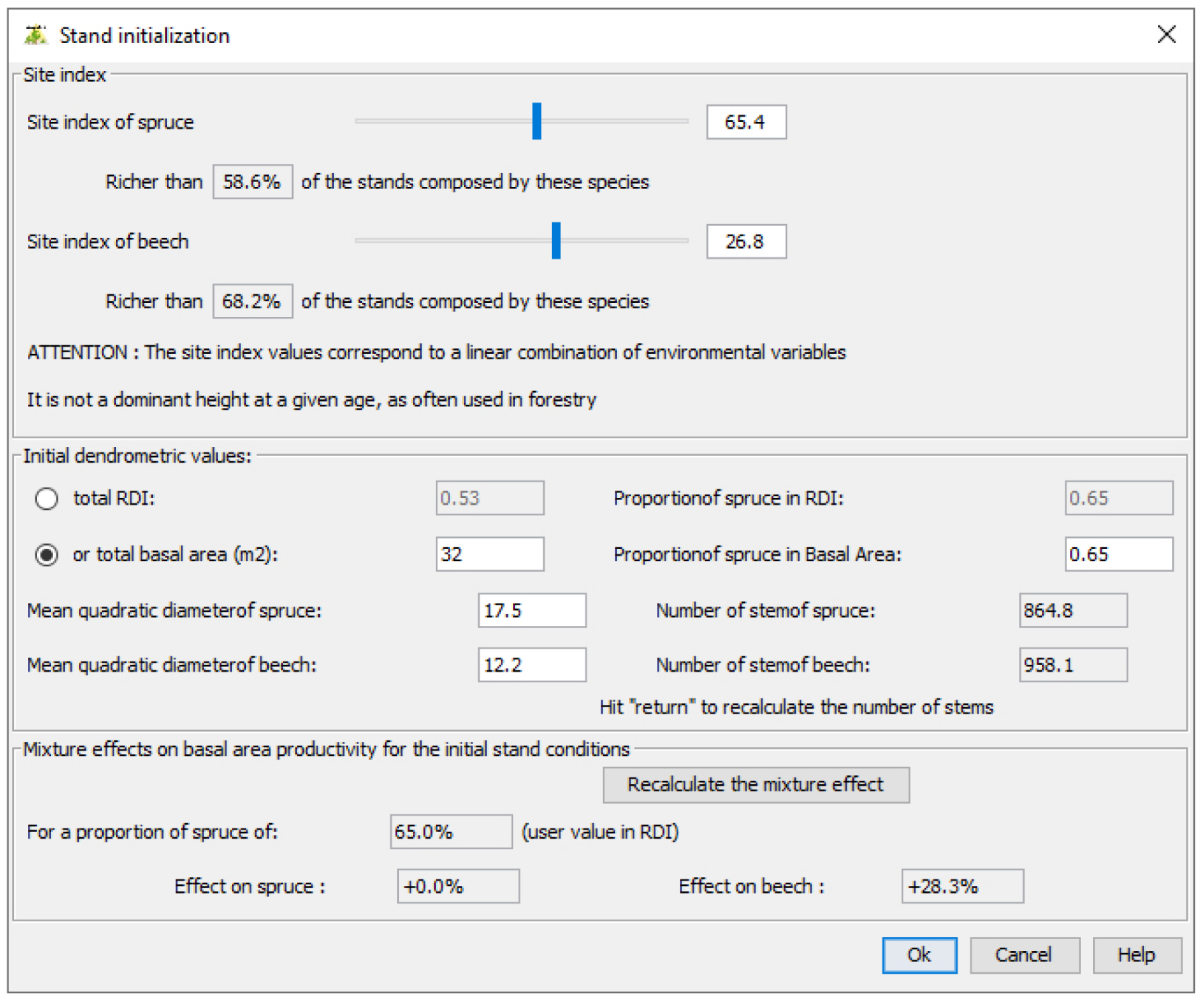
Setting site indices and initial dendrometric values.

Then, users must specify the site indices for the simulated species. These site indices are those used in our growth models and calculated at Equation 2. If users cannot provide the site indices, they can set their values using their observed distribution at French NFI plots. Here, we set the spruce site index to 65.4, a value greater than 58.6% of the site indices observed in the French NFI stands composed of spruce and beech. We set the beech site index to 26.8, a value greater than 68.2% of the site indices observed in the same stands ( Figure 7).

 In the same window, users must define the initial dendrometric values. The stand total density and the proportion represented by the first species must be provided either in terms of basal area or in terms of density index (DI as defined in Equation 1). In addition, species mean quadratic diameter must be specified. Here, we set the stand total basal area to 32 m ^2^.ha ^−1 ^and defined 17.5 cm and 12.2 cm as mean quadratic diameter for spruce and beech, respectively. In this window, the corresponding number of stems for each species and the effect of each species on the other species growth are shown.

### 4.2 Running simulations and implementing management operations

**Figure 8.**
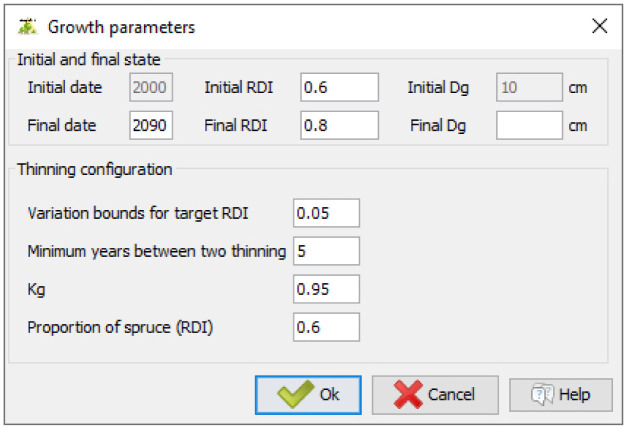
Setting thinning parameters and simulation duration.

Users must define a final date for the simulation. If they want to simulate silvicultural operations, they can set an initial and a final density (DI as defined in Equation 1; Figure 8). Instead, a final mean quadratic diameter can be provided. In that case, the simulation ends when it is reached. Here, we set the initial density to 0.6 and the final density to 0.8. In doing so, the prescribed density will gradually increase during the simulation. Variation bounds for the prescribed density must also be provided. Here, we set the variation bounds to 0.05. Thus, if the stand density exceeds the prescribed density + 0.05, then a thinning is carried out to bring the stand back to a density equal to the prescribed density - 0.05. Users must also set a minimum number of years between two thinnings and provide a Kg value (as defined in Equation 14). Here we set the minimum period between two thinnings to five years and specified *Kg* =0.95. Finally, users must specify the target proportion of the first species. Here, we set this proportion to 0.6.

### 4.3 Simulation output

**Figure 9.**
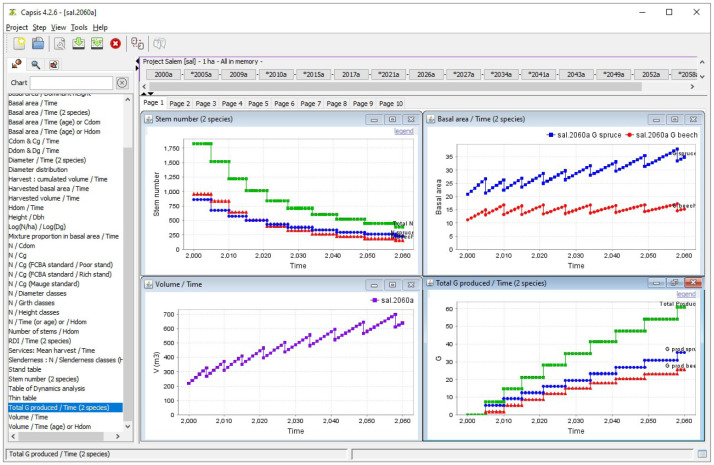
Simulation output visualisation in Capsis. The top left panel shows the total number of stems (green) as well as the number of spruce (blue) and beech (red) stems during the simulation. The top right panel shows the evolution of both species basal area. The bottom left panel shows the evolution of the stand total volume. Finally, the bottom right panel shows the total harvested basal area (green) as well as the harvested basal area of spruce (blue) and beech (red).

Simulation outputs can be directly displayed within Capsis as either graphs or tables. They can also be exported or simply copied and pasted in a spreadsheet. Figure 9 shows some outputs of the simulation performed with the parameters set in the previous sections.

### 4.4 Use cases

Here, to illustrate the mixture effect simulated in Salem, we compare the production of the spruce - beech mixed stand used as an example in the previous sections to the production of pure stands of spruce and beech. We simulated these pure stands using the same site indices, initial dendrometric values and thinning parameters as those used for our spruce - beech mixed stand. We exported these simulations in ”.txt” format and analysed them with *R*.

**Figure 10.**
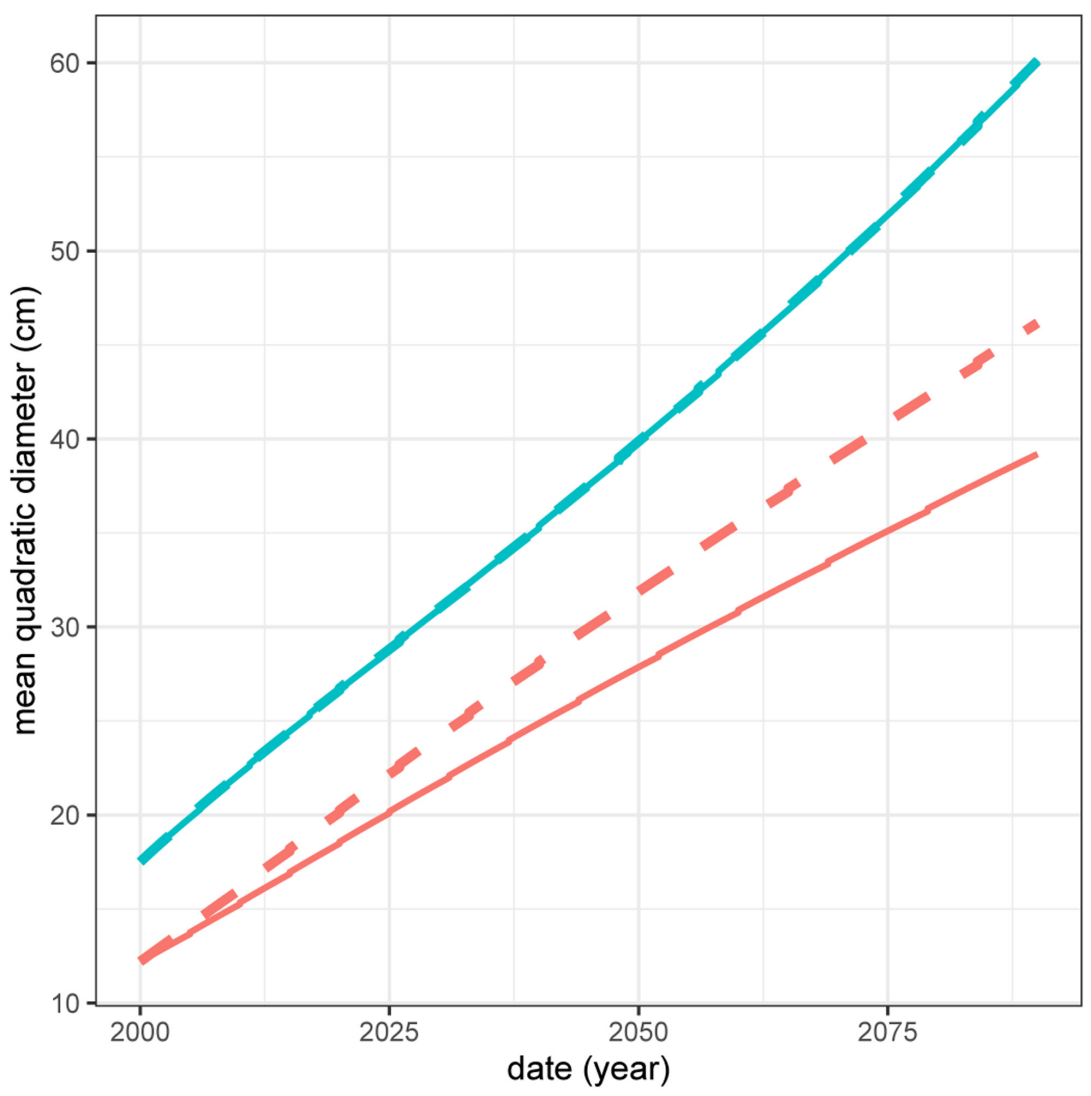
Mean quadratic diameter of beech (red) and spruce (blue) in pure (full line) and mixed (dashed line) stands.

**Figure 11.**
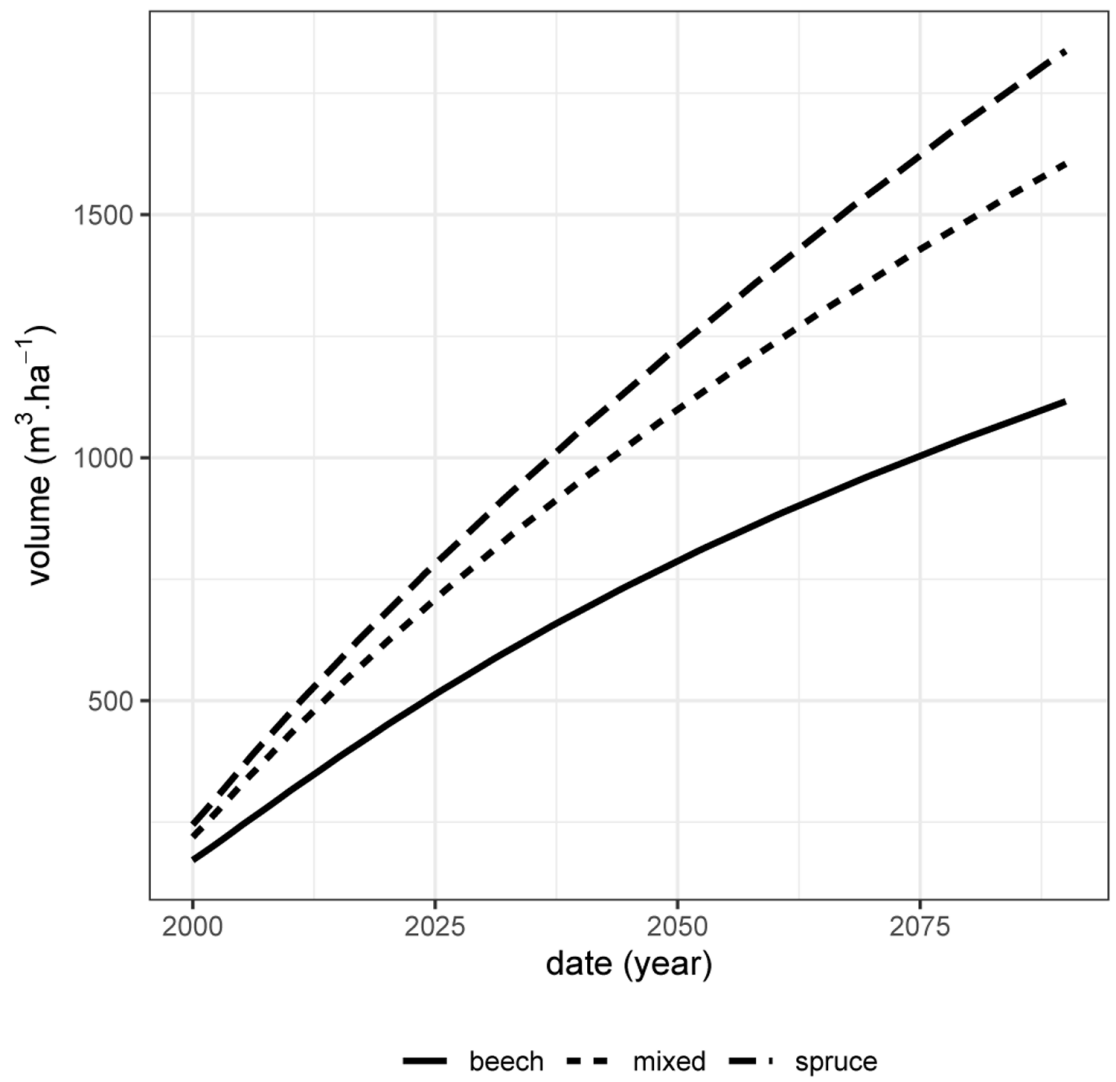
Total volume production over time (harvested volume + stand volume) for the spruce - beech mixed stand (short-dashed line), the pure spruce stand (long-dashed line) and the pure beech stand (full line). These volumes are given for 1 ha.

The positive effect of spruce on beech (see Table 6) implies that the total production of a spruce - beech mixed stand is higher than the sum of the productions of the two species in pure stands weighted by their proportion in the mixed stand. Thus, if the management objective is to produce a maximum volume of these two species, then a mixed stand is more advantageous than two separate pure stands. Here, due to the positive effect of spruce on beech, in 2090 beech trees will be 17.8% larger in diameter in the mixed stand than in the pure stand (39.2 cm in the pure stand compared to 46.2 cm in the mixed stand; Figure 10). However, despite this positive effect of spruce on beech, there is no ”transgressive overyielding” ( Pretzsch & Schütze, 2009): the pure stand of the most productive species (here spruce) remains more productive than the mixture ( Figure 11). Thus, if the only management objective is to produce as much volume as possible whatever the species, then the pure stand of spruce is the most suitable.

The mixture effect presented here cannot be generalised. As a reminder, the mixture effect depends on species assemblages and environmental factors (see Section 3.2).

## Discussion

Here, we presented the structure of Salem, a new simulator aiming at predicting the dynamics of pure and mixed even-aged stands and which makes it possible to simulate management operations. We demonstrated the functioning of Salem by simulating the dynamics and management of hypothetical stands.

In addition to provide species-specific growth and allometric models, one major outcome of Salem is the positive effect of species mixture we found on most species growth. This result is in line with a growing number of studies suggesting that the diversity - productivity relationship is most often positive in forest ecosystems ( Ammer, 2019; Liang *et al*., 2016). We also found this effect of mixture is stronger at poor sites than at richer sites. This finding is consistent with previous studies showing the benefit of species mixture on stand productivity increases as site conditions become harsher ( Paquette & Messier, 2011; Toïgo *et al*., 2015), as predicted by the stress gradient hypothesis ( Bertness & Callaway, 1994).

Like any phenomenological model, Salem might be less reliable outside its calibration range and is therefore intended to be used above all over the French territory. However, the great diversity of ecological conditions found in the French NFI dataset, which spans over four biogeographical regions (mediterranean, alpine, continental and atlantic; Roekaerts, 2002), makes Salem predictions particularly robust. In fact, some preliminary results indicate Salem remains reliable at various locations in Europe (Mats Mahnken, unpublished report). Independently from the simulator, European foresters can also consider using the species-specific models that constitute Salem. Here, we presented several dendrometrical based models for 12 widespread species in Europe: growth models including or not mixture effect, bark models, diameter distribution models, circumference - height relationship models as well as volume equations.

## Conclusion

Salem makes it possible to compare the productivity of pure and mixed stands depending on environmental conditions and according to user-defined management strategies. It can therefore help forest managers and stakeholders as well as policy makers assess the potential benefit of shifting from pure to mixed stands. Thus, Salem could facilitate the expansion of mixed stands in managed forest landscapes as recommended by international policies ( Anon, 2006; European-Environment-Agency, 2008).

## Data availability

### Underlying data

NFI data are freely available to download from: https://inventaire-forestier.ign.fr/spip.php?rubrique270. 

### Extended data

Zenodo: Salem 2.0 extended data. https://doi.org/10.5281/zenodo.4742559 ( Aussenac *et al*., 2021).

This project contains the following extended data within the file ’Salem 2 0 extended data.pdf’:

- Table A.1 (Estimates of the s _0_ parameter in Equation 10)
- Table A.2 (Estimates of the s _0_ and s _1_ parameters in Equation 11)
- Figure A.1 (Mixture effect on species growth)
- Figure A.2 (Normal distributions fitted to observed distributions of individual tree diameters by classes of mean quadratic diameter. Example for *Quercus robur*)
- Table A.3 (Mean diameter at breast heigh (dbh), height and volume of trees in the volume dataset, for species that are not relevant to Salem)
- Table A.4 (Species-specific parameter estimates of the volume models ( Equation 19), for species that are not relevant to Salem)

Data are available under the terms of the Creative Commons Attribution 4.0 International license (CC-BY 4.0).

## Software availability

Software available from: http://capsis.cirad.fr/capsis/download

Archived source code at time of publication: https://doi.org/10.5281/zenodo.4738734 ( Vallet *et al*., 2021) 

License: GNU Lesser General Public License version 2
