# Supplementary material for: The Salem simulator version 2.0: a tool for predicting the productivity of pure and mixed forest stands and simulating management operations
Source: Open Res Eur. 2021 Nov 1;1:61. Originally published 2021 Jun 4. [Version 2] doi: 10.12688/openreseurope.13671.2 (PMC10445841; doi:10.12688/openreseurope.13671.2)
Supplement: Peer review history

# Peer review: Reviewer response for version 2

**DOI:** 10.21956/openreseurope.15363.r27941

**Published:** 2021-11-16

**Authors:** Tomáš Hlásny

**Affiliations:** 1 Faculty of Forestry and Wood Sciences, Czech University of Life Sciences Prague, Prague, Czech Republic

The authors have addressed my concerns, I would like to award the article a status of Approved.

Is the rationale for developing the new software tool clearly explained?

Partly

Is the description of the software tool technically sound?

Yes

Are sufficient details of the code, methods and analysis (if applicable) provided to allow replication of the software development and its use by others?

Yes

Is sufficient information provided to allow interpretation of the expected output datasets and any results generated using the tool?

Partly

Reviewer Expertise:

NA

I confirm that I have read this submission and believe that I have an appropriate level of expertise to confirm that it is of an acceptable scientific standard.

# Peer review: Reviewer response for version 1

**DOI:** 10.21956/openreseurope.14745.r27500

**Published:** 2021-09-13

**Authors:** Tomáš Hlásny

**Affiliations:** 1 Faculty of Forestry and Wood Sciences, Czech University of Life Sciences Prague, Prague, Czech Republic

The article introduces a new simulation model aimed at the productivity of mixed forest stands. The overall idea and structure of the model are well described, and the paper is suitable for indexing subject to revision. I have three major comments:  

 In Abstract, Introduction, and Discussion – the authors suggest the improved productivity is the main motivation for the transition from monospecific to mixed stands. However, these motivations are much broader, including risk reduction (insurance hypothesis), creating more diverse habitats, etc. In my opinion, the effort to reduce the high risk of failure of monospecific stands is currently even a higher priority than fostering productivity. I suggest highlighting these aspects in the text and supporting these statements by references.

 Testing and knowing the accuracy is a crucial prerequisite for any model`s use. This aspect is, however, missing in the reviewed manuscript. I see (at least) two aspects that require rigorous testing:

1. Splitting the data into calibration and testing samples would help understand the underlying equations' generality and accuracy.
2. The authors selected one of four possible options for simulating the species mixture. It is, however, unclear, if this one is optimal. At least comparing the performance of the used approach based on multipliers with the most straightforward approach based on the weighting of pure stands would indicate if the presented solution brings the desired added value.

The authors are of course, free to come with any other testing design. However, I consider this aspect critical for both practical model`s use and research. The testing results would also indicate the ways for further model`s development.

 My final comments concern lacking consideration of changing growth and competition patterns due to climate change. The model was parameterized based on the past data, which characterize certain interactions between the concurrent species. However, climate change modifies species growth performance and interspecific interactions, making it difficult to transfer past models to the future. Although such considerations are the domain of process-based models, it is necessary to discuss this limitation.

 Further, I have some formal comments, which mainly aim to improve the text`s clarity.

 I suggest relaxing statements such as "… model that best meet the needs of forest managers". Each model is “best” is some regard, plus there is no survey of managers` needs presented in the paper to bring such a strong statement.

 Remove please the last paragraph of the introduction. Grant information is stated at the end, and there is no need to emphasize it here. Writing in the intro that Salem is mature and reliable ….. is not adequate as well. This can be stated in discussion or conclusions based on the evidence presented in the paper. The last two sentences are redundant and should be removed. Strongly recommend revising the text and removing such idle sentences.

 The last paragraph of page 3 and first paragraph of page 4 – it is written three times here that the model operates at an annual scale, I suggest simplifying the text.

 Model description

 Stands had to be regular stands – the sentence cannot be understood.

 diameter measurements and the radial growth measures. … measurement rather than measures.

 sylvoécorégions does not seem to be an English word, revise please.

 Table 6 caption – On the other hand …. probably … On the contrary.

 3.2.3 Results synthesis – I think a better title would be Demonstration, as no synthesis is provided, just an example of calculating mixture effect,

 "The mixture effect on the other species is presented in Figure A.1 of the extended data (Aussenac et al., 2021)“ – I cannot understand the meaning of citation on the end of the sentence. It should be clarified if these results were already published. I understood that parameterizing the mixture effect was done in the current study.

 Section 3.2.3 and 4.4 seem to deal with the same/similar problem (exemplifying the effect of species mixture). Check please if this structure is correct.

 The text contains a large number of idle clauses (mainly at the beginning of sentences), I am giving some examples here. Recommend authors improving the wording to increases the text`s clarity. (# … # indicates part to be removed)

 #In the first instance#, Salem requires the stand initial state as input, *i.e.* the basal area (BA), the mean quadratic diameter (Dg), and the site indices for one or two species (for mono- or bi-specific stands).

 #In Salem#, site indices are calculated from a species-specific combination of environmental variables ( Section 3.1). 

 #At that stage#, if the simulated stand density exce …

 The general functioning of Salem is summarised in Figure 1, which shows how the different models are connected to each other. -> Model structure is shown in Figure 1.

Is the rationale for developing the new software tool clearly explained?

Partly

Is the description of the software tool technically sound?

Yes

Are sufficient details of the code, methods and analysis (if applicable) provided to allow replication of the software development and its use by others?

Yes

Is sufficient information provided to allow interpretation of the expected output datasets and any results generated using the tool?

Partly

Reviewer Expertise:

Ecosystem modelling, climate change impacts and adaptation, disturbance dynamics and management. I am not a model developer to comment adequately on implementation details

I confirm that I have read this submission and believe that I have an appropriate level of expertise to confirm that it is of an acceptable scientific standard, however I have significant reservations, as outlined above.

# Response

**Published:** 2021-10-22

**Authors:** Raphaël Aussenac

**Affiliations:** CIRAD, France

Dear reviewer,

 Thank you for your comments. In summary, in this new version we discuss the limitations of Salem regarding the reliability of its predictions under the future climate. We also point out that forest managers may want to shift from pure to mixed stands not only for a productivity gain but also to reduce risks against disturbances. We also made some minor changes to the text to improve its clarity. Please find below our replies to your comments.  

***In Abstract, Introduction, and Discussion – the authors suggest the improved productivity is the main motivation for the transition from monospecific to mixed stands. However, these motivations are much broader, including risk reduction (insurance hypothesis), creating more diverse habitats, etc. In my opinion, the effort to reduce the high risk of failure of monospecific stands is currently even a higher priority than fostering productivity. I suggest highlighting these aspects in the text and supporting these statements by references.***

 We agree with the reviewer, risk reduction may be a reason to shift from monospecific stands to mixed stands. However, we did not work at all on this aspect, Salem only focuses on the effect of mixture on species growth. We believe the point raised by the reviewer is worth mentioning in the abstract and introduction, but should probably not be mentioned in the discussion, as our work does not shed any light on this issue.

 We modified the first paragraph of the abstract as follows: “A growing body of research suggests mixed-species stands are generally more productive than pure stands as well as less sensitive to disturbances. However, these effects of mixture depend on species assemblages and environmental conditions. Here, we present the Salem simulator, a tool that can help forest managers assess the potential benefit of shifting from pure to mixed stands from a productivity perspective. Salem predicts the dynamics of pure and mixed even-aged stands and makes it possible to simulate management operations. Its purpose is to be a decision support tool for forest managers and stakeholders as well as for policy makers. It is also designed to conduct virtual experiments and help answer research questions.”  

 We modified the first paragraph of the introduction as follows: “Over the last decades, mixed-species stands have received growing attention. From the perspective of forest managers, they have a number of advantages compared to monospecific stands. They can be more productive (Ammer, 2019; Liang et al., 2016) as well as less sensitive to various disturbances such as windstorms (Schütz et al. 2006), pathogen invasions (Haas et al. 2011) or insect outbreaks (Jactel & Brockerhoff 2007; Castagneyrol et al. 2013; Klapwijk & Björkman 2018). However, these effects of mixture are highly variable and depend on species and environmental conditions. As a result, forest managers often lack information to consider mixed stands as a possible alternative to pure stands. Forest dynamics models ( Pretzsch, 2009; Weiskittel et al., 2011) can help fill this information gap. In this article, we will focus on the potential benefit of shifting from pure to mixed stands from a productivity perspective, we will not cover the aspect of risk reduction against disturbances.”  

***Testing and knowing the accuracy is a crucial prerequisite for any model`s use. This aspect is, however, missing in the reviewed manuscript. I see (at least) two aspects that require rigorous testing:***

***Splitting the data into calibration and testing samples would help understand the underlying equations' generality and accuracy.***

 We agree with the reviewer, testing and evaluating models is essential before any use. However, we have deliberately chosen not to include an evaluation in this paper which is already 29 pages long, so as not to make it any longer and because the evaluation work is part of a forthcoming publication. Indeed, we have carried out a multi-partner study in which we evaluated the predictions of 13 models (including Salem) against observed data from across Europe (from Spain to Scandinavia), within the ISIMIP project (https://www.isimip.org/). Salem is ranked n°1 for the accuracy of its predictions. Following the ORE guidelines, we alluded to this work in our paper (p.25): « […] some preliminary results indicate Salem remains reliable at various locations in Europe (Mats Mahnken, unpublished report) ». In this forthcoming publication, the evaluation dataset is completely independent from the calibration dataset, which makes the evaluation particularly robust. Splitting the data into calibration and testing samples as suggested by the reviewer would not be as robust for two main reasons: (1) being calibrated on less data, Salem will lose some of its predictive power, and (2) the calibration and testing samples are not completely independent, they are linked by the NFI protocol. The fact that 13 models are being jointly evaluated also helps better apprehend the relative quality of Salem's predictions. Following a discussion with the editor, this evaluation work may be passed on to the reviewer upon request.

***The authors selected one of four possible options for simulating the species mixture. It is, however, unclear, if this one is optimal. At least comparing the performance of the used approach based on multipliers with the most straightforward approach based on the weighting of pure stands would indicate if the presented solution brings the desired added value.***

 The comparison proposed by the reviewer is already implicitly provided. In fact, the mixture effect on a species growth is calculated as the deviation from the species expected growth in pure stand weighted by its proportion (see equation 12 and 13). Thus, if a species has a significant effect on the growth of another species (s0 significant in Table 6), this means that the multiplier approach we use brings some added value. As mentioned in our previous reply, 13 models are being jointly evaluated in the forthcoming publication. These models use a wide variety of modelling approaches. We believe this forthcoming evaluation of Salem is far more comprehensive than any evaluation we could carry out here, in particular, to compare modelling approaches. We would rather not present an incomplete or less comprehensive evaluation of Salem in this paper.

***The authors are of course, free to come with any other testing design. However, I consider this aspect critical for both practical model`s use and research. The testing results would also indicate the ways for further model`s development.***

 See replies to the two previous comments  

***My final comments concern lacking consideration of changing growth and competition patterns due to climate change. The model was parameterized based on the past data, which characterize certain interactions between the concurrent species. However, climate change modifies species growth performance and interspecific interactions, making it difficult to transfer past models to the future. Although such considerations are the domain of process-based models, it is necessary to discuss this limitation.***

 We agree with the reviewer and included this point in the paragraph dealing with Salem limitations in the discussion. The paragraph now reads as follows: “A major limitation of Salem is due to its phenomenological nature which implies that its predictions may be less reliable as we move further away from its calibration range. Salem is therefore intended to be used above all over the French territory. Similarly, Salem being calibrated on the past climate, the validity of its predictions in the future remains uncertain. However, the great diversity of ecological and climate conditions found in the French NFI dataset, which spans over four biogeographical regions (mediterranean, alpine, continental and atlantic; Roekaerts, 2002), could make Salem predictions robust over a larger area and in a wide range of climate conditions. In fact, some preliminary results indicate Salem remains reliable at various locations in Europe (Mats Mahnken, unpublished report). In the forthcoming version of Salem under development, the effect of climate fluctuations both on species growth and on the mixture effect (i.e. on species interactions) will be modelled. This integration of climate should strengthen the quality of Salem predictions under the future climate. The method has already been developed and applied to a case study (Vallet & Pérot, 2018).”

***Further, I have some formal comments, which mainly aim to improve the text`s clarity.*** ***I suggest relaxing statements such as "… model that best meet the needs of forest managers". Each model is “best” is some regard, plus there is no survey of managers` needs presented in the paper to bring such a strong statement.***

 We agree with this comment and removed this statement.

***Remove please the last paragraph of the introduction. Grant information is stated at the end, and there is no need to emphasize it here. Writing in the intro that Salem is mature and reliable ….. is not adequate as well. This can be stated in discussion or conclusions based on the evidence presented in the paper. The last two sentences are redundant and should be removed. Strongly recommend revising the text and removing such idle sentences.***

 As suggested, we removed the part of the paragraph redundant with the grant information section and the mention to Salem reliability and maturity. We revised the rest of the paragraph which now reads as follows: “We present the general functioning of Salem in Section 2. We then provide details on the different models used in Section 3. Finally, we show how to simulate stands with Salem and how to implement management operations in Section 4.”

***The last paragraph of page 3 and first paragraph of page 4 – it is written three times here that the model operates at an annual scale, I suggest simplifying the text.***

 We could not find any mention to the annual time step in the last paragraph of page 3 and first paragraph of page 4 in the pdf. The reviewer may be referring to the last paragraph of page 4 and first paragraph of page 5. We removed the first mention to the annual time step to simplify the text, as it is indeed redundant with the end of the sentence. The two remaining mentions, however, seem to us important to keep.

***Model description*** ***Stands had to be regular stands – the sentence cannot be understood.***

 Replaced by: “Stands had to be classified as even-aged stands by the French NFI.”

***diameter measurements and the radial growth measures. … measurement rather than measures.***

 Modified as suggested.

***sylvoécorégions does not seem to be an English word, revise please.***

 This word is immediately defined in the parenthesis that follows it. We have italicised it to emphasise that it is not an English word and we have removed its French acronym from the definition, as it is not needed to understand the word and may bring some confusion.

***Table 6 caption – On the other hand …. probably … On the contrary*** **.**

 Modified as suggested.

***3.2.3 Results synthesis – I think a better title would be Demonstration, as no synthesis is provided, just an example of calculating mixture effect.***

 This section consists of two paragraphs. In the first one, we present as an example the results we obtained for fir, i.e. the effects on the growth of fir of different species depending on site productivity. In the second paragraph, we summarise the results obtained for all species (but see also reply to following comment). These are original results derived from the models we present in this article. We would therefore rather keep ‘results synthesis’ as title for this section. Maybe the last sentence of the first paragraph was not in the right place. We moved it to the beginning of the second paragraph. This might help better understand the section. The second paragraph therefore begins as follows: “The mixture effect on the other species is presented in Figure A.1 of the supplementary information (see extended data; Aussenac et al., 2021). Overall, we found the mixture effect on species growth was most often positive…”

   

***"The mixture effect on the other species is presented in Figure A.1 of the extended data (Aussenac et al., 2021)“ – I cannot understand the meaning of citation on the end of the sentence. It should be clarified if these results were already published. I understood that parameterizing the mixture effect was done in the current study.***

 We follow here the guidelines of ORE: supplementary information must be deposited in an approved repository and cited as extended data in the main body of the article. We therefore refer here to original results that derive from the models we present in this study. The content of the extended data is summarised in the ‘Data availability’ section as required by ORE guidelines. To avoid any confusion we slightly modified the way we refer to the extended data throughout the document using this formulation: “Table XX of the supplementary information (see extended data; Aussenac et al. 2021)”.

**Section 3.2.3 and 4.4 seem to deal with the same/similar problem (exemplifying the effect of species mixture). Check please if this structure is correct.**

*Hopefully our replies to the two previous comments have clarified the purpose of section 3.2.3: summarising the results of our models regarding the effect of mixture on species growth. As for Section 4.4, it exemplifies the effect of mixture in a stand parameterised from Salem graphical user interface. These two sections are therefore not redundant.*

***The text contains a large number of idle clauses (mainly at the beginning of sentences), I am giving some examples here. Recommend authors improving the wording to increases the text`s clarity. (# … # indicates part to be removed)** **#In the first instance#, Salem requires the stand initial state as input, i.e. the basal area (BA), the mean quadratic diameter (Dg), and the site indices for one or two species (for mono- or bi-specific stands).** **#In Salem#, site indices are calculated from a species-specific combination of environmental variables (Section 3.1).** **#At that stage#, if the simulated stand density exce …** **The general functioning of Salem is summarised in Figure 1, which shows how the different** **models are connected to each other. -> Model structure is shown in Figure 1.***

 On the contrary, we believe that these carefully chosen formulations help understand the text. This has been confirmed to us by native English speakers who have read it. Since the preference for one writing style or another is rather subjective and since this comment is only a recommendation we would rather keep these formulations.

# Peer review: Reviewer response for version 1

**DOI:** 10.21956/openreseurope.14745.r27123

**Published:** 2021-07-22

**Authors:** Thomas Knoke

**Affiliations:** 1 Institute of Forest Management, TUM School of Life Sciences Weihenstephan, Technische Universität München, Freising, Germany

Disclaimer:

 The paper reports numerous statistical formulas and coefficients. It was impossible for the reviewer to check the validity of each formula and coefficient. However, I have studied the paper carefully and searched for any possible inconsistencies from the Methods description and the Results interpretation.

 "The Salem simulator version 2.0: a tool for predicting the productivity of pure and mixed stands and simulating management operations" is a very useful tool to support the analysis and management of mixed forests in France. The paper has great merits in providing basic dendrometric information about a large number of mixed forests, while these stand types comprise two tree species. The simulation model Salem can reproduce the main existing results on mixing effects, such as overyielding. It is site specific so that locally adapted simulations are possible. The database is extensive. In sum, Salem provides a very helpful tool to design successful mixed stand types and to predict likely outcomes when managing mixed forests.

 Further comments:

- One could consider to make sure that the units of the variables are always indicated, when such variables are used (e.g. for all Tables).
- It would perhaps be advisable to include that "mortality" does not cover stand failure by events, such as storm, drought or barck beetles.
- Cores from how many trees have been taken?
- Has the self-thinning line been parameterized only from untreated plots?
- Is the use of the word circumference appropriate? In the equations we find only diameters.

Is the rationale for developing the new software tool clearly explained?

Yes

Is the description of the software tool technically sound?

Yes

Are sufficient details of the code, methods and analysis (if applicable) provided to allow replication of the software development and its use by others?

Yes

Is sufficient information provided to allow interpretation of the expected output datasets and any results generated using the tool?

Yes

Reviewer Expertise:

Forest science, forest economics, optimization

I confirm that I have read this submission and believe that I have an appropriate level of expertise to confirm that it is of an acceptable scientific standard.

# Response

**Published:** 2021-10-22

**Authors:** Raphaël Aussenac

**Affiliations:** CIRAD, France

Dear reviewer,

 Thank you for your comments. In summary, in this new version we discuss the limitations of Salem regarding the reliability of its predictions under the future climate. We also point out that forest managers may want to shift from pure to mixed stands not only for a productivity gain but also to reduce risks against disturbances. We also made some minor changes to the text to improve its clarity. Please find below our replies to your comments.

***One could consider to make sure that the units of the variables are always indicated, when such variables are used (e.g. for all Tables).***

 We added a missing unit in Figure 4 legend.

***It would perhaps be advisable to include that "mortality" does not cover stand failure by events, such as storm, drought or barck beetles.***

 We added two sentences in section 2 (Implementation) to clarify this point: “The only mortality factor simulated in Salem is therefore competition. Mortality due to natural disturbances such as storms, droughts or insect outbreaks is not simulated.”

***Cores from how many trees have been taken?***

 Overall 52543 cores were collected in pure stands and 33747 cores in mixed stands. We added these figures in the legends of Table 2 (Number and features of inventory plots in pure stands for each species) and Table 5 (Number of inventory plots for each species pair).

***Has the self-thinning line been parameterized only from untreated plots?***

 The species-specific self-thinning boundaries were parameterised using NFI data collected in pure stands. A wide variety of stands is therefore represented in the data. However, as mentioned in section 3.1.2, we used a quantile regression to fit these self-thinning boundaries. The quantile of the regression was 0.95, meaning that the resulting line corresponds to the 5% of the denser stands of the dataset. In Table 1 we specify the data used for the calibration of the self-thinning boundaries and refer the reader to Toïgo et al. (2018) for further details on the method. Subsequently, in section 3.1.2 we briefly present the calibration method and the calibration results. We would rather not expand further on this subject so as not to make this paper, which is already 29 pages long, any longer.

***Is the use of the word circumference appropriate? In the equations we find only diameters.***

 We confirm that the word circumference is appropriate wherever it is used. We use this word in section 3.6 (Models of individual tree height) or in reference to this section, and circumference does appear in our height models (parameter c130 in equation 18, Table 9 and Table 10). Both circumference and diameter were used throughout the document because the Salem simulator is a compilation of works that have been carried out for several years and in different projects.
